# Face masks influence emotion judgments of facial expressions: a drift–diffusion model

**DOI:** 10.1038/s41598-023-35381-4

**Published:** 2023-05-31

**Authors:** W. Craig Williams, Eisha Haque, Becky Mai, Vinod Venkatraman

**Affiliations:** 1 Department of Marketing, Fox School of Business, Temple University, Philadelphia, PA 19122 USA; 2 Department of Psychology, University of Toronto, Toronto, Canada

**Keywords:** Human behaviour, Viral infection

## Abstract

Face masks slow the spread of SARS-CoV-2, but it has been unknown how masks might reshape social interaction. One important possibility is that masks may influence how individuals communicate emotion through facial expressions. Here, we clarify to what extent—and how—masks influence *facial emotion* communication, through drift–diffusion modeling (DDM). Over two independent pre-registered studies, conducted three and 6 months into the COVID-19 pandemic, online participants judged expressions of 6 emotions (anger, disgust, fear, happiness, sadness, surprise) with the lower or upper face “masked” or unmasked. Participants in Study 1 (*N* = 228) correctly identified expressions above chance with lower face masks. However, they were less likely—and slower—to correctly identify these expressions relative to without masks, and they accumulated evidence for emotion more slowly—via decreased drift rate in DDM. This pattern replicated and intensified 3 months later in Study 2 (N = 264). These findings highlight how effectively individuals still communicate with masks, but also explain why they can experience difficulties communicating when masked. By revealing evidence accumulation as the underlying mechanism, this work suggests that time-sensitive situations may risk miscommunication with masks. This research could inform critical interventions to promote continued mask wearing as needed.

## Introduction

Since the COVID-19 pandemic began, people around the world started wearing face masks as a simple and effective way to reduce the spread of the SARS-CoV-2 virus. Face masks decrease the amount of coronavirus RNA that individuals exhale^1^ and mandating masks reduces community spread of SARS-CoV-2^2,3^. Masks play an essential role in the global response to COVID-19 even as vaccines are distributed^4^, and individuals will likely wear masks in response to future pandemics and seasonal influenza viruses, coronaviruses, and rhinoviruses. Given their significance for public health, sudden prevalence, and likely staying power, it is critical to better understand how masks impact human social interaction worldwide^5^.

One important way face masks may reshape social life is by influencing how individuals communicate emotion through facial expressions. Facial expressions are one of several key channels for conveying emotions to other people^6^. Clear facial emotion communication tracks important outcomes across real-world contexts, from patients experiencing better outcomes after interacting with their doctors^7^, to customers perceiving employees as more warm and reporting greater intention to buy from them^8^. More broadly, emotion communication scaffolds social life by facilitating cooperation^9^, learning^10^, and the development of new relationships^11^. Masking facial expressions could therefore have wide-ranging consequences for emotion communication and social interaction across diverse settings^12^.

Face masks could impact multiple distinct aspects of how individuals perceive facial expressions. Masks may influence how accurately and how quickly individuals judge facial expressions, and underlying these decision outcomes, masks may further influence how rapidly individuals accumulate evidence for making emotion judgments. For example, individuals may fail to identify masked disgust expressions, and they may be slower to identify these expressions, because they accumulate evidence for disgust more slowly with masks. This evidence accumulation—or *drift rate*—is a key decision-making process that explains differences in both response accuracy and response speed. When individuals accumulate evidence more rapidly, they respond more accurately and give faster correct responses, whereas when individuals accumulate evidence less rapidly, they respond less accurately and give slower correct responses^13,14^. For instance, as individuals learn decision-making tasks, they perform more accurately and quickly because they accumulate evidence more rapidly^15^. Drift rates are estimated using diffusion modeling techniques such as drift–diffusion modeling (DDM)^14,16,17^, which has been used to model diverse types of judgments^18^, such as financial^19^, dietary^20^, altruistic^21^, punitive^22^ and emotional^23^.

Face masks could impact how individuals identify emotion expressions in general or how they identify certain expressions in particular. On the one hand, individuals gaze at both the eyes and the mouth of diverse expressions^24–26^. On the other hand, individuals gaze at these features—and use information from them^27–29^—to varying degrees for different expressions. Concealing the lower or upper face—as with face masks or sunglasses—could thus have idiosyncratic consequences for emotion communication. For instance, masks may interfere with how individuals communicate disgust, but not anger, whereas sunglasses may interfere with communicating anger, but not disgust.

Research clarifying how face masks impact emotion communication could inform interventions to promote mask wearing and facilitate global behavioral change in response to COVID-19 and future pandemics^30^. Many individuals report not wearing masks because they experience difficulties communicating when masked^31,32^. Recent work has shown that individuals are less accurate judging masked facial expressions including in adults^33–37^, in children^38–40^, and across cultures^41^. While accuracy is one important aspect of facial emotion communication, little research has examined whether masks may also influence the speed of this communication, or underlying processes such as evidence accumulation. Such research may assist interventions aimed at promoting mask wearing in at least two ways. First, it could educate the public about the degree to which masks influence emotion communication. Second, if evidence accumulation was the mechanism underlying mask effects over decision accuracy and speed, it would point to model-based strategies that individuals may use to compensate for the effects of masks—such as slowing down to accumulate more evidence. Conversely, if masks influenced speed or accuracy without impacting evidence accumulation, then it could point to other potential mechanisms and strategies to compensate for mask effects.

**Figure 1:**
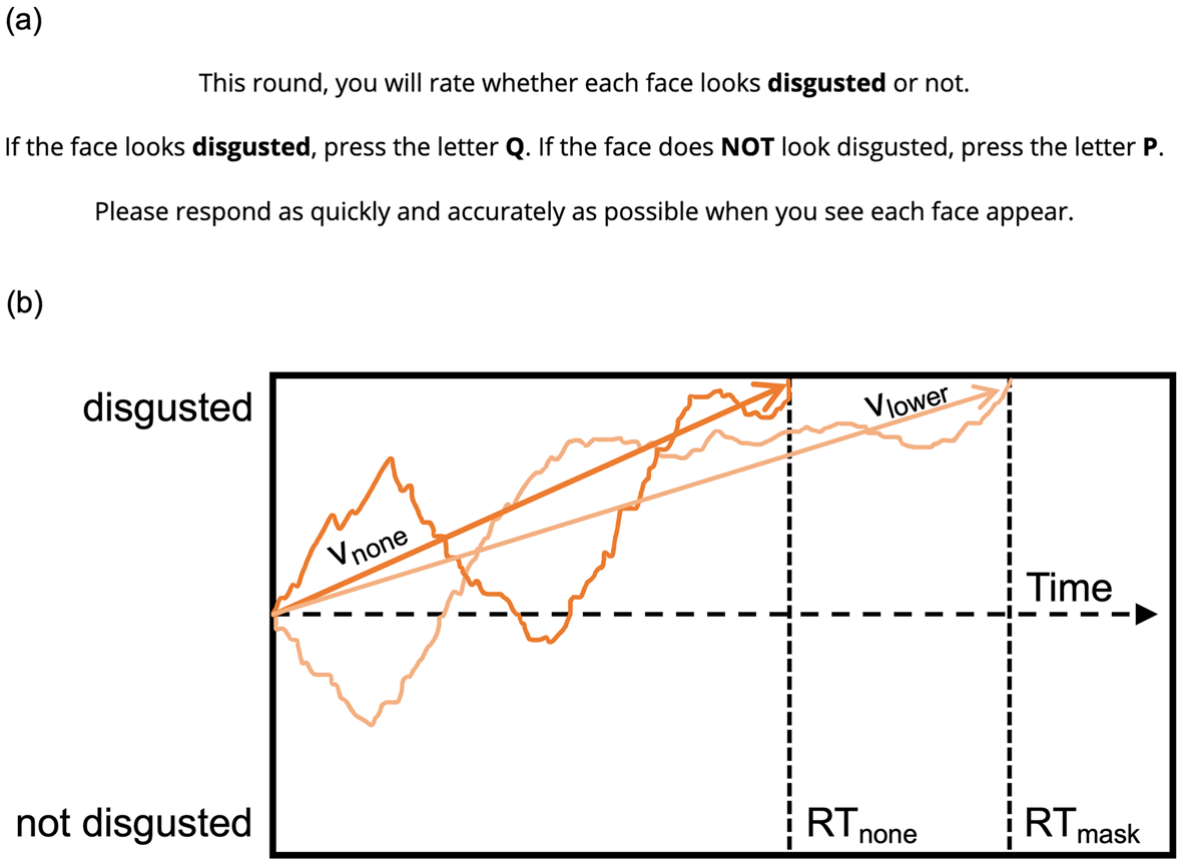
Emotion judgment task and modeling. (**a**) Sample task instructions. On each round, participants rated whether or not faces showed one of six emotions (anger, disgust, fear, happiness, sadness, surprise) by pressing a key with either their left or right hand (key pairing randomly assigned by participant). Emotion rating type was randomized across task rounds. (**b**) Hypothetical drift–diffusion model (DDM). This example shows how rapidly participants accumulate evidence for disgust—as indicated by *drift rates*—from disgusted expressions without masks (v_none_) versus disgusted expressions with lower masks (v_lower_). Here, steeper slopes reflect faster evidence accumulation—via more positive drift rates.

**Figure 2:**
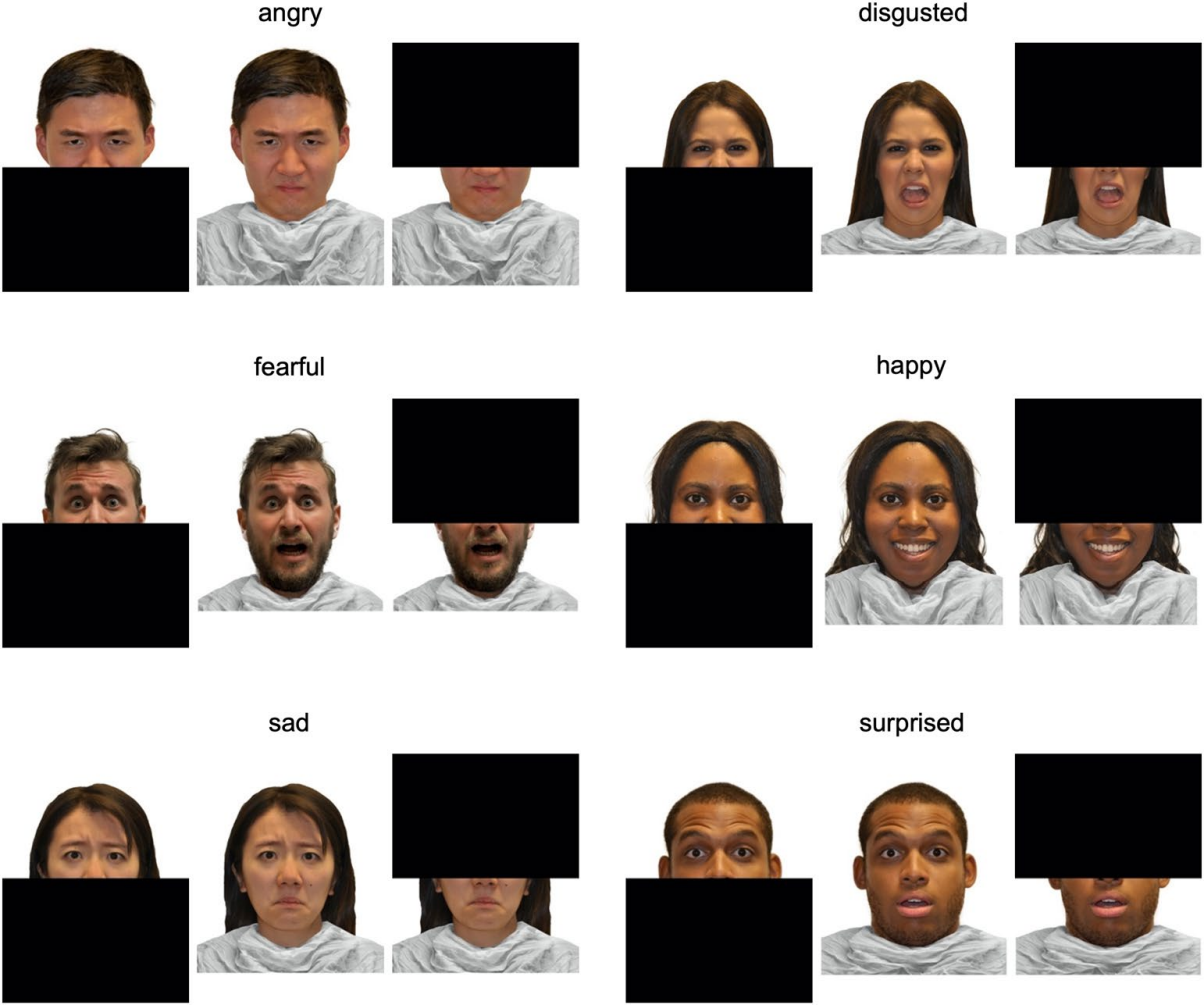
Sample expressions with masks. Participants viewed facial expressions of 6 emotions (anger, disgust, fear, happiness, sadness, surprise) with 3 types of “face masks” (lower, none, upper). On each round, participants viewed one of six blocks of faces, with blocks fully counterbalanced for emotion expressions and masks, and faces presented in random order. Every block included one expression from each actor with all three types of masks. Participants in Study 2 viewed an ethnically diverse set of female and male East Asian, Black, Hispanic, and white faces drawn from the racially diverse affective expression (RADIATE) face stimulus set.

Here, we examined how masks influence emotion expression judgments over two independent pre-registered studies (Study 1 *N* = 228, Study 2 *N* = 264) conducted three and 6 months into the COVID-19 pandemic. Participants in both studies completed 6 rounds of viewing and rating facial expressions of 6 emotions (anger, disgust, fear, happiness, sadness, surprise; within-subjects) with 3 types of “face masks” (lower, upper, none; within-subjects). On each round, participants made binary ratings as quickly and accurately as possible of whether or not faces showed one of the 6 emotions (Fig. 1). Lower “masks” blacked-out faces from the tip of the nose down, whereas upper “masks” blacked-out faces from the nose-tip up (Fig. 2). Here, we prioritized experimental control of whether or not each half of the face was visible^42^—allowing us to directly compare lower versus upper masks.

This design enabled us to test whether individuals are (i) less likely to judge expressions correctly—via increased errors, (ii) slower to judge expressions correctly—via slower response times, and (iii) slower to accumulate evidence for emotion judgments—via slower drift rates, when judging expressions with masks versus without them (Fig. 1). Critically, by manipulating both lower and upper “masks,” we tested whether masks shape emotion judgments by hiding the mouth in particular, or by simply hiding one half of the face. This manipulation further provided basic insights into how the lower and upper face each contribute information towards distinct emotion judgments. Finally, by conducting Study 2 three months after Study 1, we were able to test whether the effects of masks diminished as individuals interacted more often with other individuals wearing masks, or whether these effects intensified as they interacted with other individuals less often due to social distancing.

## Methods

### Participants

We recruited participants from Amazon Mechanical Turk for two studies conducted in July and October of 2020 (Study 1 total *N* = 300; Study 2 total *N* = 300). These target total sample sizes were pre-registered through AsPredicted prior to collecting any data and pre-determined to generate high statistical power with the repeated measures designs, as informed by power analysis of Study 1 prior to Study 2 (see “Power analysis” under “Data analysis”). Data collection ceased once we reached these target total sample sizes. We collected 300 complete responses for Study 1 and 290 complete responses for Study 2. Ten incomplete responses to Study 2 were not recorded. Participants were at least 18 years of age (Study 1: M = 38.3 years, SD = 11.5; Study 2: M = 37.3 years, SD = 11.0) and they represented a diverse sample in terms of gender (Study 1: 37.7% female; Study 2: 42.8% female) and ethnicity (Study 1: 22.4% non-white; Study 2: 29.2% non-white). We obtained informed consent from all participants and all experiments were approved by the Temple University Institutional Review Board and performed in accordance with their guidelines and regulations.

### Procedure and design

Experiments were developed using the jsPsych JavaScript library^43^, which measures response times with high precision and reliability comparable to lab-based software^44,45^. Participants completed six rounds of viewing and rating facial expressions of 6 emotions (anger, disgust, fear, happiness, sadness, surprise; within-subjects) with 3 types of “face masks” (lower, upper, none; within-subjects). Participants viewed a different block of faces each round, counterbalanced for the 6 expressions and 3 masks, and rated whether or not faces expressed one of 6 emotions (anger, disgust, fear, happiness, sadness, surprise; within-subjects). Blocks of faces and types of emotion ratings were randomized across task rounds and participants viewed faces in random order within each round. On each trial, participants viewed a centered fixation cross for 1000 ms, followed by a face with the tip of the nose centered to the preceding fixation cross. Participants rated whether or not each face expressed a given emotion as quickly and accurately as possible by pressing a key with either their left or right hand (Fig. 1). Response options were randomly assigned to the left and right hands for each participant. Prior to starting each round of trials, participants were required to pass instruction checks confirming that they knew which emotion they would rate that round and which key corresponded to each response option.

### Stimuli

Six blocks of faces were fully counterbalanced for emotion expressions and “face masks” such that participants made all 6 ratings of all 6 expressions with all 3 masks. We selected 36 white female and male actors from the Radboud Faces Database for Study 1^46^ and 18 East Asian, Black, Hispanic, and white female and male actors from the Racially Diverse Affective Expression (RADIATE) face stimulus set for Study 2^47^. To create lower and upper masks, faces were split in half at the tip of the nose and either the lower half or the upper half of each face image was blacked-out (Fig. 2). This generated 648 total faces for Study 1 and 324 total faces for Study 2. Each block of faces included one expression from each actor, with all three masks, presented in random order for 108 faces per block in Study 1 and 54 faces per block in Study 2. Critically, this design counterbalanced blocks of faces for expressions and masks, as well as for actors, actors’ gender, and in Study 2, actors’ ethnicity.

### Data analysis

#### Power analysis

To determine a target total sample size that would generate high statistical power in Study 2, we conducted a power analysis of Study 1 in G*Power^48^. Here, we examined participants’ emotion ratings on trials where they could have made false negative errors—that is, trials where they viewed expressions congruent with the emotion they rated. For each expression, we selected the mask vs. no mask contrast with the largest effect (lower: disgust, happiness, sadness, surprise; upper: anger, fear) and we calculated odds ratios by taking the exponent of the corresponding *b* values (Table 1). Power analysis revealed that a target final *N* = 230 would achieve 95% power for detecting five of these six effects in two-tailed binomial logistic regression models (α = 0.05). We pre-registered this target final sample size and a target total *N* = 300 based on the rate of failed attention checks in Study 1.

**Table 1:** Failing to identify expressions: Face mask effects over emotion ratings.

	Study 1			Study 2		
	b	CI	z	b	CI	z
All faces						
Lower mask > none	− 0.76	[− 0.92, − 0.60]	− 9.16***	− 1.11	[− 1.37, − 0.86]	− 8.68***
Lower mask > upper	− 0.036	[− 0.21, 0.14]	− 0.42	− 0.50	[− 0.77, − 0.23]	− 3.57***
Upper mask > none	− 0.73	[− 0.89, − 0.56]	− 8.72***	− 0.60	[− 0.86, − 0.35]	− 4.70***
Angry faces						
Lower mask > none	0.083	[− 0.28, 0.44]	0.45	− 0.16	[− 0.48, 0.14]	− 1.05
Lower mask > upper	1.62	[1.29, 1.95]	9.43***	1.21	[0.94, 1.48]	8.83***
Upper mask > none	− 1.55	[− 1.93, − 1.18]	− 8.34***	− 1.39	[− 1.67, − 1.12]	− 9.86***
Disgusted faces						
Lower mask > none	− 1.59	[− 1.83, − 1.35]	− 12.96***	− 1.64	[− 2.16, − 1.17]	− 6.38***
Lower mask > upper	− 1.39	[− 1.66, − 1.12]	− 11.15***	− 1.33	[− 1.84, − 0.79]	− 4.74***
Upper mask > none	− 0.18	[− 0.44, 0.061]	− 1.45	− 0.29	[− 0.76, 0.21]	− 1.11
Fearful faces						
Lower mask > none	− 0.63	[− 0.88, − 0.37]	− 4.89***	− 0.75	[− 1.36, − 0.15]	− 2.61**
Lower mask > upper	0.84	[0.59, 1.07]	6.85***	0.35	[− 0.23, 0.91]	1.17
Upper mask > none	− 1.47	[− 1.73, − 1.21]	− 11.44***	− 1.12	[− 1.67, − 0.53]	− 3.93***
Happy faces						
Lower mask > none	− 0.76	[− 1.07, − 0.46]	− 4.84***	− 1.20	[− 1.70, − 0.72]	− 4.57***
Lower mask > upper	− 0.54	[− 0.85, − 0.24]	− 3.36***	− 1.11	[− 1.61, − 0.61]	− 4.15***
Upper mask > none	− 0.23	[− 0.54, 0.10]	− 1.40	− 0.062	[− 0.58, 0.44]	− 0.23
Sad faces						
Lower mask > none	− 1.24	[− 1.56, − 0.92]	− 7.70***	− 1.65	[− 2.09, − 1.16]	− 6.89***
Lower mask > upper	− 0.81	[− 1.10, − 0.50]	− 5.11***	− 1.33	[− 1.90, − 0.78]	− 5.01***
Upper mask > none	− 0.43	[− 0.74, − 0.088]	− 2.63**	− 0.29	[− 0.76, 0.23]	− 1.21
Surprised faces						
Lower mask > none	− 1.10	[− 1.42, − 0.78]	− 6.54***	− 1.78	[− 2.50, − 0.98]	− 4.76***
Upper mask > none	− 0.21	[− 0.53, 0.092]	− 1.28	− 1.13	[− 2.00, − 0.32]	− 2.66**
Upper mask > none	− 0.88	[− 1.20, − 0.53]	− 5.24***	− 0.67	[− 1.39, 0.078]	− 1.80

*p < 0.05. **p < 0.01. ***p < 0.001. *CI* = 0.95.

#### Exclusions

For both studies, we pre-registered that we would exclude participants who failed attention checks, resulting in the exclusion of 72 participants from Study 1 (final *N* = 228) and 26 participants from Study 2 (final *N* = 264). We also pre-registered that we would exclude trials with response times less than 100 ms^49^. Hierarchical drift–diffusion models required further excluding trials with response times in the top 0.5% of all trials (> 9303 ms in Study 1 and > 5737 ms in Study 2).

#### Statistics

We pre-registered the following analyses testing whether masks influenced how accurately participants rated expressions, how quickly they rated expressions, and how rapidly they accumulated evidence for rating expressions. Analyses of false negative errors examined trials where participants viewed emotion expressions congruent with the emotion they judged that round—that is, trials where participants either correctly identified expressions (e.g. rating disgust expressions as disgusted) or failed to correctly identify them (e.g. rating disgust expressions incorrectly as not disgusted). We analyzed emotion ratings, response times, and drift rates, for all 6 congruent pairings of expressions and judgments. In contrast, analyses of false positive errors examined trials where participants viewed expressions incongruent with the emotion they judged that round—that is, trials where participants either correctly distinguished between expressions (e.g. rating disgust expressions as not angry) or misidentified them (e.g. rating disgust expressions incorrectly as angry). We analyzed emotion ratings for all 30 incongruent pairings of expressions and judgments. When masks influenced these ratings, we further analyzed response times for that incongruent pairing, and when masks influenced these response times, we further analyzed drift rates for that pairing.

Analyses of emotion ratings tested the main effects of masks, collapsing across expressions, and within each type of emotion expression. Emotion ratings were analyzed through two-tailed mixed effect binomial logistic regression models including participant intercepts as random effects. Analyses of response times tested the interaction between masks and rating accuracy, as well as the simple main effects of masks for correct and incorrect ratings, collapsing across expressions and within each expression type. Response times were analyzed through two-tailed mixed effect linear regression models including participant intercepts as random effects. Response times were log-transformed to correct for their inherent skewness and flipped (i.e. multiplied by − 1) for plotting purposes. Regression coefficients and 95% confidence intervals were estimated through bootstrapped regression analyses using 1,000 iterations^50^.

Analyses of drift rates tested the main effects of masks, across expressions overall and within each expression type (Fig. 1). Drift rates were estimated through hierarchical Bayesian estimation of drift–diffusion model parameters (HDDM) using Markov chain Monte Carlo (MCMC) sampling methods^14,16,17^. These models estimated posterior distributions of regression coefficients for the effects of masks over drift rate (v) parameters and included participant intercepts as random effects. In addition, these models also estimated parameters for response bias (z), boundary separation (a), non-decision time (t), and inter-trial variability in all parameters (sz, sa, st, sv), as covariates averaging over mask conditions. Parameter coefficients and 95% credible intervals were estimated by drawing 5000 samples from posterior distributions and discarding the initial 200 ‘burn-in’ samples. Bayesian hypothesis testing compared the posterior distributions of drift rate coefficients across mask conditions and estimated the probability that they differed from each other and the probability that they differed from 0. Follow-up analyses additionally tested the effects of masks over boundary separation (a) controlling for mask effects over drift rate (v).

### Preprint

Preprint posted to *PsyArXiv* under Creative Commons Attribution 4.0 International Public License: https://doi.org/10.31234/osf.io/a8yxf.

## Results

Over two sets of pre-registered analyses, we tested the influence of lower and upper masks over how accurately participants rated expressions, how quickly they made these ratings, how rapidly they accumulated evidence for emotion from expressions, and how much evidence they required to make these judgments. Here, we report analyses of false negative errors—that is, trials where participants viewed expressions congruent to the emotion they judged, and could have failed to correctly identify expressions—for emotion ratings, RTs, drift rates, and boundary separations (see Supplementary Note for analyses of false positive errors).

### False negative errors

#### Emotion ratings

Analyses of emotion ratings compared how accurately participants identified expressions with lower and upper masks versus chance (50%), versus expressions without masks, and versus one another, as increasingly conservative tests of emotion rating accuracy. Participants in Study 1 correctly identified all 6 types of expressions above chance with lower masks, and expressions of disgust, happiness, sadness, and surprise, but not anger or fear, with upper masks.

**Figure 3:**
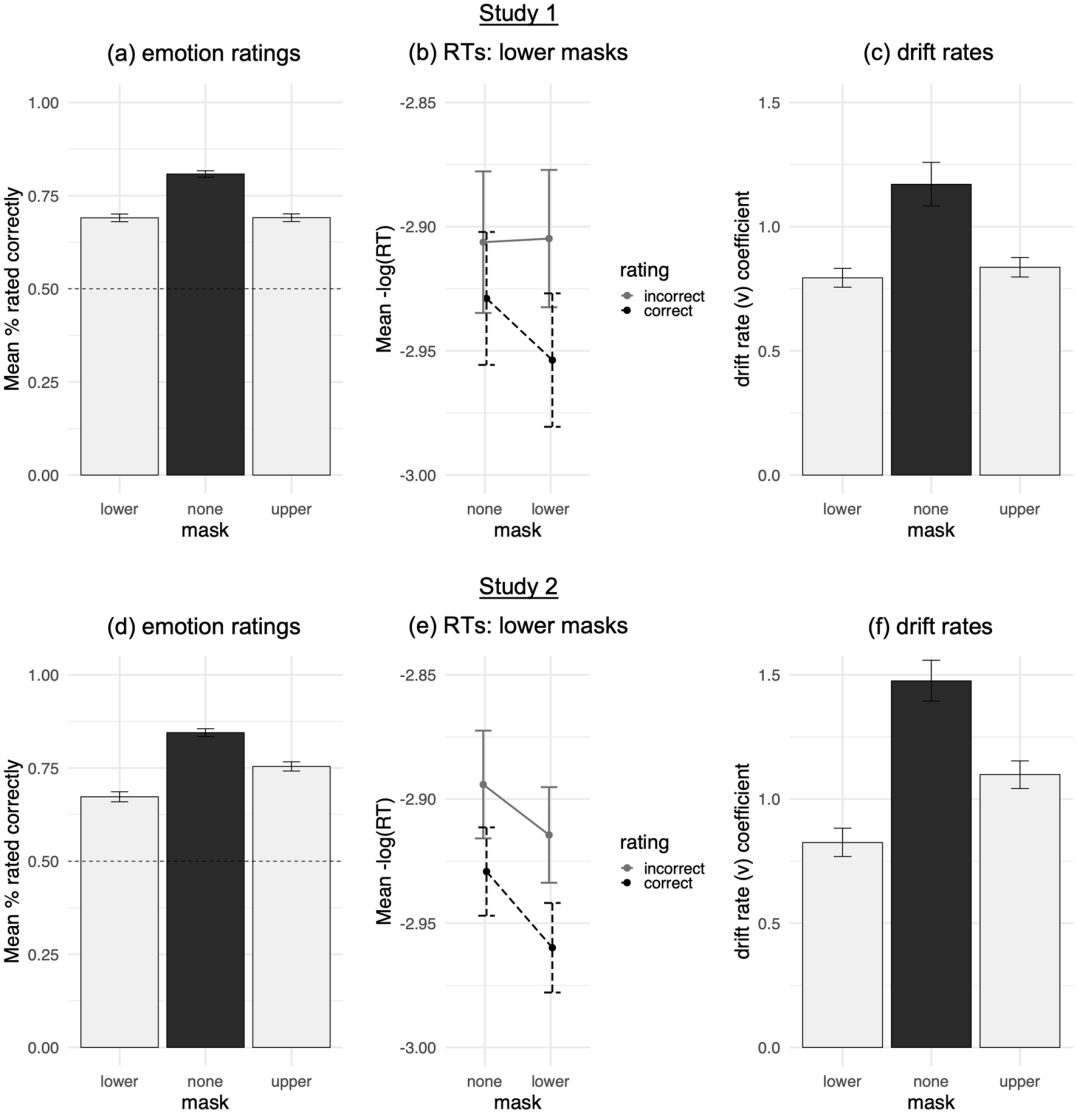
Correct judgments vs. false negative errors. Effects of masks over (**a**,**d**) emotion ratings whereby higher mean values reflect increased accuracy, (**b**,**e**) emotion rating RTs (flipped; lower masks vs. no masks) whereby higher mean values reflect faster responses, and (**c**,**f**) drift rate coefficients whereby higher coefficient values reflect increased drift rate towards correct judgments. Error bars depict 95% confidence intervals (**a**,**b**,**d**,**e**) and 95% credible intervals (**c**,**f**).

**Figure 4:**
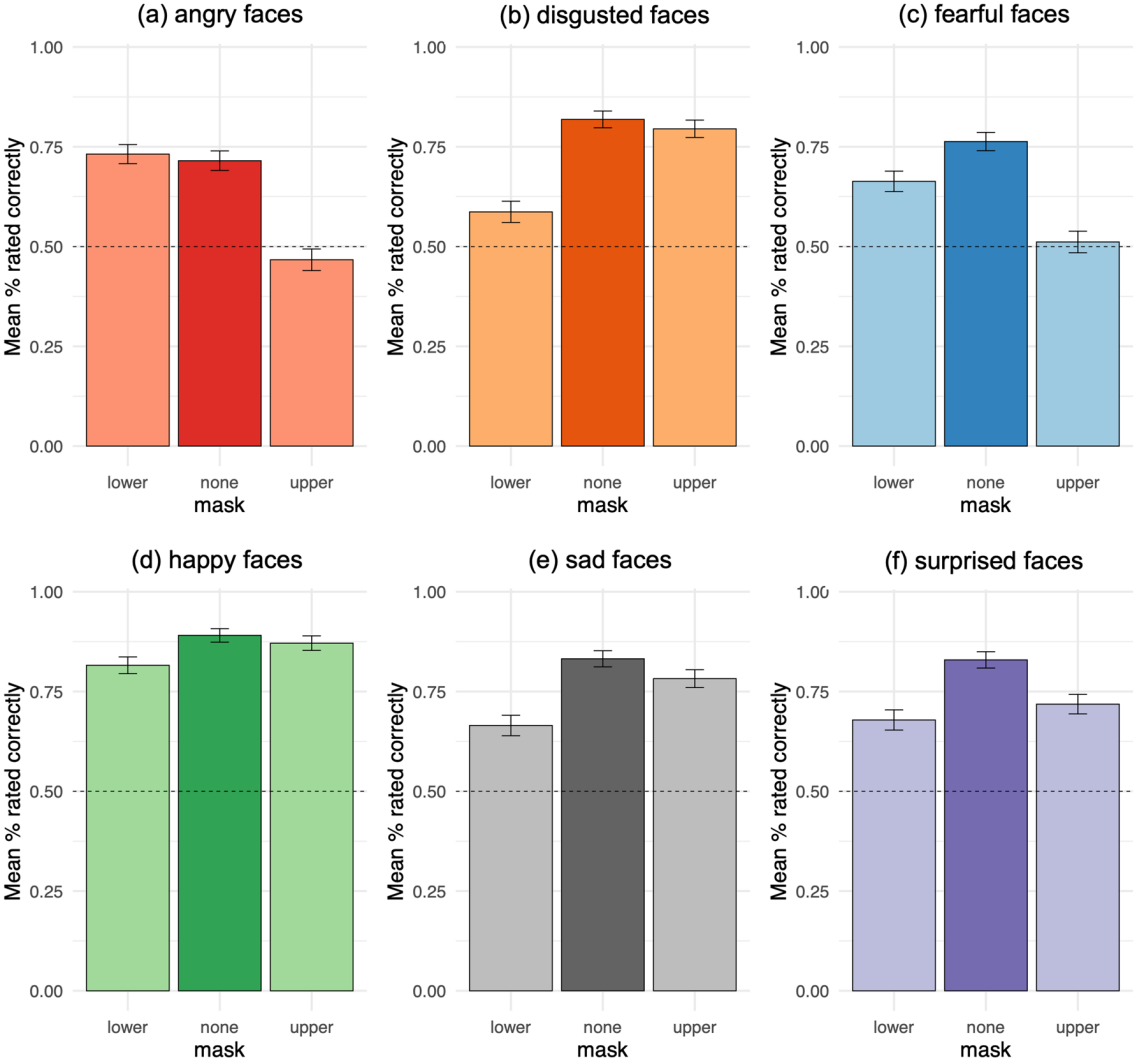
Mean emotion ratings by masks: correct ratings vs. false negative errors rating (**a**) angry, (**b**) disgusted, (**c**) fearful, (**d**) happy, (**e**) sad, and (**f**) surprised faces (Study 1). Higher mean values reflect increased accuracy and decreased false negative errors. Error bars depict 95% confidence intervals.

However, participants were less accurate identifying expressions with lower and upper masks than without masks, collapsing across emotions (Fig. 3, Table 1). With lower masks, participants were less accurate identifying expressions of disgust, fear, happiness, sadness, and surprise, but not anger, relative to without masks (Fig. 4). With upper masks, they were less accurate identifying expressions of anger, fear, sadness, and surprise, but not disgust or happiness.

Participants did not significantly differ in their accuracy identifying expressions overall with lower versus upper masks (Table 1). Nonetheless, participants were less accurate identifying disgusted, happy, and sad expressions with lower masks versus with upper masks (Fig. 4). By contrast, they were less accurate identifying angry and fearful expressions with upper masks as compared to lower masks.

#### Emotion rating RTs

Response time analyses compared how quickly participants made correct and incorrect ratings of expressions with lower and upper masks, versus expressions without masks and versus each other, to assess emotion rating speed. Collapsing across emotions, participants were slower to correctly identify expressions with lower masks (Mdn = 903.66 ms, SD = 818.85), but not upper masks (Mdn = 866.65 ms, SD = 741.91), relative to without masks (Mdn = 838.78 ms, SD = 734.29), as revealed by an interaction between mask conditions and emotion ratings for lower masks only (Fig. 3).

**Figure 5:**
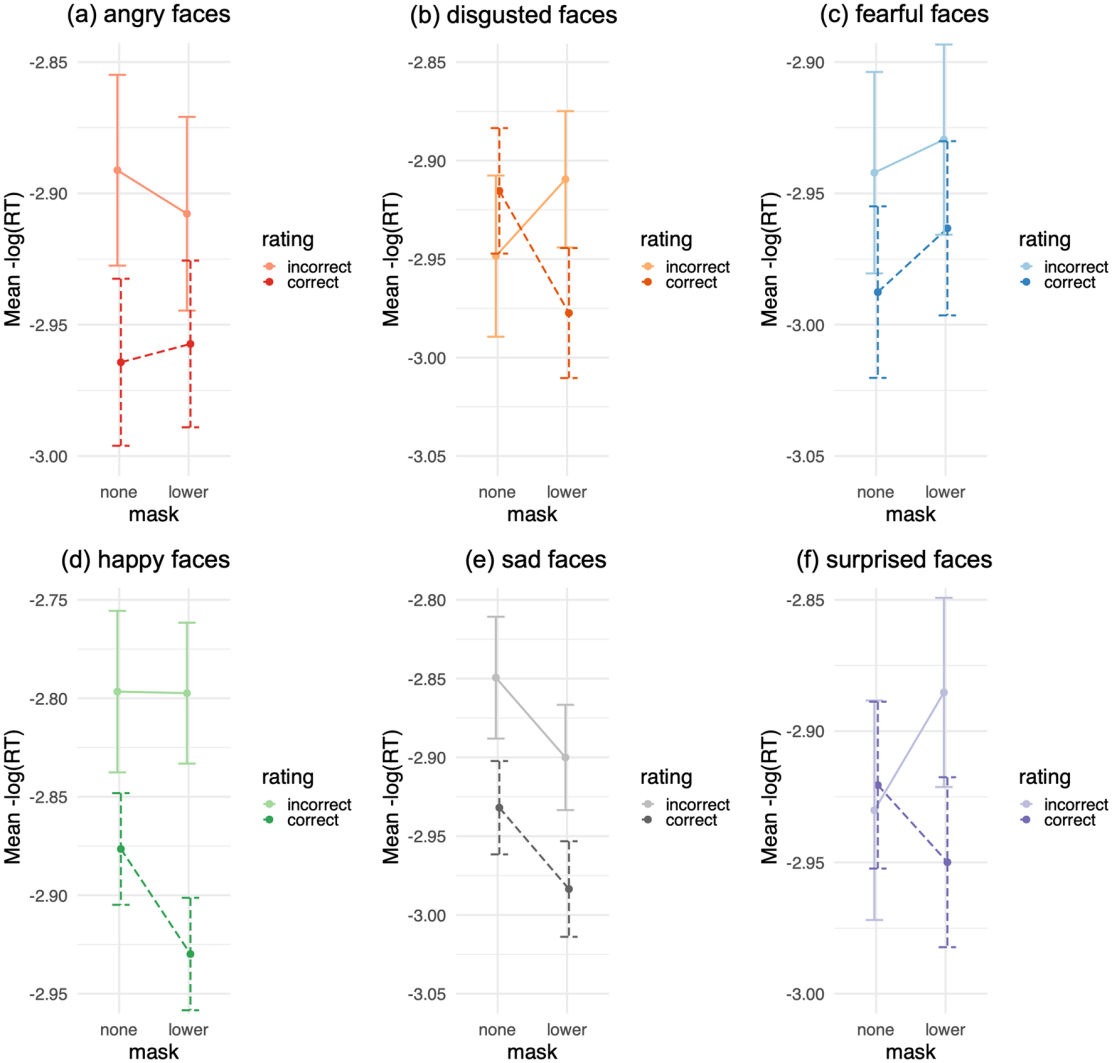
Mean emotion rating RTs (flipped) by masks and emotion ratings: correct rating RTs vs. false negative error RTs rating (**a**) angry, (**b**) disgusted, (**c**) fearful, (**d**) happy, (**e**) sad, and (**f**) surprised faces, with lower masks vs. no masks (Study 1). Higher mean values reflect faster responses. Error bars depict 95% confidence intervals.

With lower masks, participants were slower to correctly identify disgust, happiness, sadness, and surprise than without masks, and as shown by interactions for disgust, happiness, and surprise (Fig. 5, Table 2). In contrast, they were faster to correctly identify fear with lower masks, versus without masks, and they showed no significant difference for anger. With upper masks, participants were slower to correctly identify anger and surprise, but no other expressions, than without masks, and as revealed by an interaction between masks and emotion ratings for surprise (Supplementary Fig. S1).

**Table 2:** Failing to identify expressions: Lower face mask effects over emotion rating RTs.

	Study 1			Study 2		
	b	CI	t	b	CI	t
All faces						
Face mask * rating	− 0.026	[− 0.040, − 0.011]	− 3.22**	− 0.010	[− 0.029, 0.01]	− 1.10
Correct: lower > none	− 0.026	[− 0.034, − 0.019]	− 7.07***	− 0.032	[− 0.040, − 0.024]	− 7.97***
Incorrect: lower > none	0.00	[− 0.017, 0.016]	− 0.015	− 0.016	[− 0.037, 0.004]	− 1.64
Angry faces						
Face mask * rating	0.024	[− 0.014, 0.059]	1.28	0.045	[− 0.012, 0.10]	1.48
Correct: lower > none	0.006	[− 012, 0.023]	0.66	0.017	[− 0.001, 0.036]	1.69
Incorrect: lower > none	− 0.023	[− 0.060, 0.016]	− 1.21	− 0.048	[− 0.012, 0.026]	− 1.24
Disgusted faces						
Face mask * rating	− 0.10	[− 0.15, − 0.062]	− 5.11***	− 0.047	[− 0.090, − 0.003]	− 2.04*
Correct: lower > none	− 0.066	[− 0.084, − 0.049]	− 7.73***	− 0.066	[− 0.091, − 0.039]	− 5.22***
Incorrect: lower > none	0.029	[− 0.012, 0.072]	1.43	− 0.016	[− 0.049, 0.020]	− 0.84
Fearful faces						
Face mask * rating	0.012	[− 0.023, 0.047]	0.65	0.022	[− 0.021, 0.061]	1.00
Correct: lower > none	0.024	[0.005, 0.040]	2.72**	− 0.019	[− 0.033, − 0.002]	− 2.39*
Incorrect: lower > none	0.009	[− 0.025, 0.044]	0.49	− 0.035	[− 0.088, 0.021]	− 1.22
Happy faces						
Face mask * rating	− 0.053	[− 0.093, − 0.008]	− 2.50*	− 0.048	[− 0.093, − 0.005]	− 2.27*
Correct: lower > none	− 0.051	[− 0.066, − 0.038]	− 7.37***	− 0.072	[− 0.086, − 0.058]	− 9.63***
Incorrect: lower > none	− 0.007	[− 0.061, 0.051]	− 0.25	− 0.018	[− 0.060, 0.026]	− 0.79
Sad faces						
Face mask * rating	0.00	[− 0.039, 0.037]	− 0.050	− 0.031	[− 0.075, 0.012]	− 1.34
Correct: lower > none	− 0.054	[− 0.070, − 0.039]	− 6.71***	− 0.061	[− 0.079, − 0.044]	− 6.17***
Incorrect: lower > none	− 0.062	[− 0.10, − 0.017]	− 3.01**	− 0.034	[− 0.084, 0.013]	− 1.39
Surprised faces						
Face mask * rating	− 0.074	[− 0.11, − 0.035]	− 3.60***	− 0.015	[− 0.057, 0.026]	− 0.68
Correct: lower > none	− 0.030	[− 0.046, − 0.015]	− 3.56***	− 0.028	[− 0.044, − 0.009]	− 3.18**
Incorrect: lower > none	0.033	[− 0.016, 0.083]	1.35	− 0.023	[− 0.071, 0.029]	− 0.90

*p < 0.05. **p < 0.01. ***p < 0.001. *CI* = 0.95.

Participants were also slower to correctly identify expressions with lower masks versus with upper masks across emotions overall, and as indicated by an interaction (Supplementary Table S2). In particular, they were slower to correctly identify disgusted, happy, and sad expressions with lower masks, versus with upper masks, and as revealed by an interaction for disgusted expressions (Supplementary Fig. S2). Conversely, they were slower to correctly identify angry and fearful expressions with upper masks in comparison to with lower masks.

#### Emotion rating drift rates

Analyses of drift rates compared how rapidly participants accumulated evidence for emotion from expressions with lower and upper masks versus null (0) drift rate—indicating evidence accumulation towards correct or incorrect judgments on average—as well as versus expressions without masks, and versus one another, as increasingly conservative tests of evidence accumulation rate. Participants accumulated evidence towards correctly identifying all 6 types of expressions with lower and upper masks, as indicated by greater than null (0) drift rates, all P(lower/upper mask < 0) < 0.0001.

**Figure 6:**
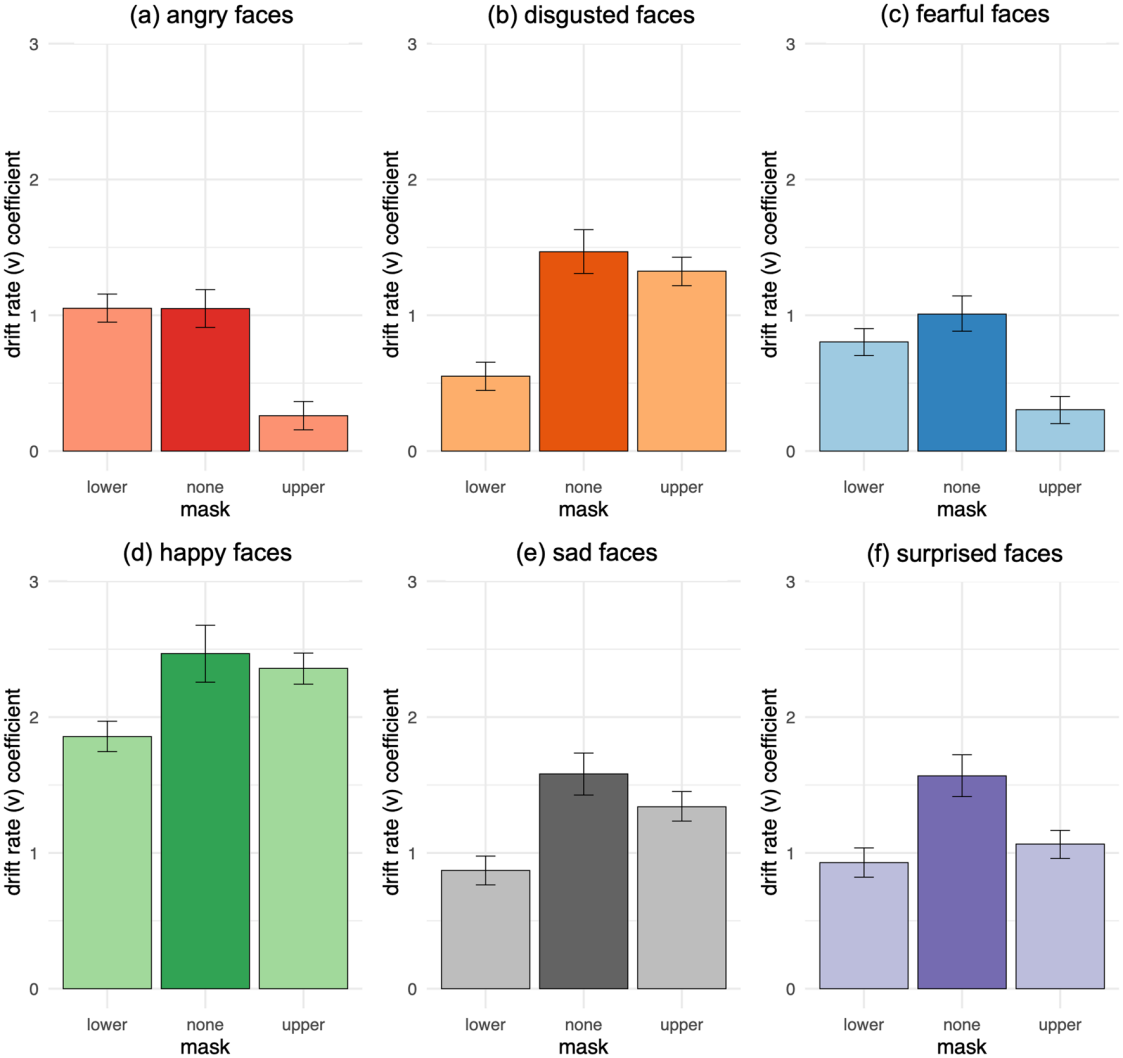
Drift rate coefficients by masks: drift rate towards correct judgments vs. false negative errors rating (**a**) angry, (**b**) disgusted, (**c**) fearful, (**d**) happy, (**e**) sad, and (**f**) surprised faces (Study 1). Higher coefficient values reflect increased drift rate towards correct judgments and decreased drift rate towards false negative errors. Error bars depict 95% credible intervals.

However, consistent with their decreased accuracy and speed, participants accumulated evidence more slowly from expressions with lower and upper masks than from expressions without masks, collapsing across emotions (Fig. 3). With lower masks, participants accumulated evidence for disgust, fear, happiness, sadness, and surprise more slowly than without masks, all P(lower mask > none) < 0.0001; but not anger (Fig. 6). With upper masks, they accumulated evidence for anger, fear, sadness, surprise, all P(upper mask > none) < 0.0001; disgust, P(upper mask > none) = 0.0029; and happiness, P(upper mask > none) = 0.029; more slowly than without masks.

Participants also accumulated evidence more slowly from expressions with lower masks, as compared to upper masks, collapsing across emotions, P(lower mask > upper mask) = 0.017 (Fig. 3). Specifically, they accumulated evidence more slowly from expressions of disgust, happiness, sadness, all P(lower mask > upper mask) < 0.0001; and surprise, P(lower mask > upper mask) = 0.0052; with lower masks relative to upper masks (Fig. 6). By contrast, participants accumulated evidence for anger and fear more slowly from expressions with upper masks as compared to lower masks, both P(upper mask > lower mask) < 0.0001.

#### Emotion rating boundary separations

Boundary separation analyses similarly compared how much evidence participants required for judgments with lower and upper masks versus without masks. Collapsing across emotions, participants required less evidence when identifying expressions with upper masks (*b* = 1.72, 95% CI [1.70, 1.74]) than without masks (*b* = 1.77, 95% CI [1.71, 1.84]), P(upper mask > none) < 0.0001); but showed no significant difference with lower masks versus without masks.

With upper masks, participants required less evidence for identifying fear (*b* = 1.70, 95% CI [1.65, 1.76]) than without masks (*b* = 1.80, 95% CI [1.72, 1.88]), P(upper mask > none) = 0.0002; but did not show a significant difference in how much evidence they required for identifying anger, disgust, happiness, sadness, or surprise. With lower masks, participants required less evidence than without masks for fear (lower mask: *b* = 1.69, 95% CI [1.64, 1.75]; none: *b* = 1.80, 95% CI [1.72, 1.88]; P(lower mask > none) = 0.0002); but showed no significant difference for anger, disgust, and surprise. Conversely, they required *more* evidence than without masks for happiness (lower mask: *b* = 1.79, 95% CI [1.73, 1.84]; none: *b* = 1.67, 95% CI [1.57, 1.77]; P(lower mask < none) < 0.0001) and sadness (lower mask: *b* = 1.72, 95% CI [1.67, 1.77]; none: *b* = 1.67, 95% CI [1.58, 1.75]; P(lower mask < none) = 0.027).

Collapsing across emotions, participants required more evidence for identifying expressions with lower masks (*b* = 1.78, 95% CI [1.76, 1.81]) relative to upper masks (*b* = 1.72, 95% CI [1.70, 1.74]), P(lower mask < upper mask) < 0.0001. This effect only held however for identifying happiness with lower masks (*b* = 1.79, 95% CI [1.73, 1.84]) in comparison to upper masks (*b* = 1.65, 95% CI [1.59, 1.70]), P(lower mask < upper mask) < 0.0001. Participants showed no significant difference in the evidence required for identifying anger, disgust, fear, sadness, or surprise.

#### Study 1 summary

Participants in Study 1 rated expressions above chance overall with both lower and upper masks and they accumulated evidence in favor of identifying masked expressions correctly on average. However, participants were worse at perceiving expressions of disgust, happiness, sadness, and surprise with lower masks relative to without masks, and worse at perceiving expressions of anger and surprise with upper masks as compared to without masks—as indicated by decreased accuracy, speed, and rate of evidence accumulation. Similarly, participants were also worse at judging disgusted, happy, and sad expressions with lower versus upper masks, and worse at judging angry and fearful expressions with upper versus lower masks—via reduced accuracy, speed, and rate of evidence accumulation.

#### Study 2 summary

Participants in Study 2 closely replicated this pattern 3 months later when judging an ethnically diverse set of facial expressions (see Supplementary Note for analyses of Study 2 false negative errors). They also rated expressions above chance with lower and upper masks, and accumulated evidence in favor of correctly identifying these expressions. Nonetheless, they too were worse at judging all expressions except anger with lower masks as compared to without masks, and worse at judging expressions of anger and fear with upper masks relative to without masks—as reflected by decreased accuracy, speed, and drift rate. These participants were likewise worse at perceiving disgusted, happy, and sad expressions with lower versus upper masks, and worse at perceiving angry expressions with upper versus lower masks.

#### False negative errors over the COVID-19 pandemic

These influences of masks intensified over 3 months, as participants showed stronger effects of lower masks—but not upper masks—at 6 months into the COVID-19 pandemic in Study 2, versus 3 months in Study 1. Collapsing across emotions, participants were slower to accumulate evidence for emotion from expressions with lower masks—relative to expressions without masks—in Study 2 (*b* =  − 0.65, 95% CI [− 0.71, − 0.59]) versus in Study 1 (*b* =  − 0.38, 95% CI [− 0.41, − 0.34]), P(lower mask_2_ − none_2_ > lower mask_1_ – none_1_) < 0.0001 (Fig. 3). This pattern held for expressions of fear (Study 2: *b* =  − 0.42, 95% CI [− 0.57, − 0.27]; Study 1: b =  − 0.21, 95% CI [− 0.31, − 0.11]; P(lower mask_2_—none_2_ > lower mask_1_ – none_1_) = 0.0075), happiness (Study 2: *b* =  − 1.12, 95% CI [− 1.28, − 0.95]; Study 1: *b* =  − 0.61, 95% CI [− 0.72, − 0.50]; P(lower mask_2_—none_2_ > lower mask_1_ – none_1_) < 0.0001), sadness (Study 2: *b* =  − 1.12, 95% CI [− 1.27, − 0.97]; Study 1: *b* =  − 0.71, 95% CI [− 0.82, − 0.60]; P(lower mask_2_—none_2_ > lower mask_1_ – none_1_) < 0.0001), and surprise (Study 2: b =  − 0.91, 95% CI [− 1.07, − 0.76]; Study 1: b =  − 0.64, 95% CI [− 0.75, − 0.53]; P(lower mask_2_—none_2_ > lower mask_1_ – none_1_) = 0.0019); but not anger or disgust. Conversely, participants showed no significant differences across studies in evidence accumulation for expressions with upper masks—relative to expressions without masks—for any of the six emotions, or when collapsing across emotions.

### False positive errors

Masks also influenced how participants distinguished between expressions (see Supplementary Note for analyses of false positive errors). With lower masks, relative to without masks, participants in Study 1 were more likely to misperceive surprised expressions as angry, sad expressions as surprised, and all expressions except fear as happy—as reflected by decreased accuracy, speed, and rate of evidence accumulation. However, they were also less likely to misperceive fearful expressions as disgusted or sad with lower masks as compared to without masks—via increased accuracy, speed, and drift rate. With upper masks, in comparison to without masks, participants were less likely to misperceive surprised expressions as fearful, fearful expressions as surprised, disgusted expressions as angry, angry expressions as disgusted, and angry and sad expressions as fearful—via increased accuracy, speed, and drift rate.

Study 2 replicated many of these effects after 3 months with participants judging an ethnically diverse set of faces. With lower masks, as compared to without masks, participants were more likely to misjudge surprised expressions as angry, angry expressions as surprised, sad expressions as happy, happy expressions as sad, and surprised expressions as sad—as shown by decreased accuracy, speed, and rate of evidence accumulation. However, they were less likely to misjudge angry expressions as sad, and disgusted expressions as surprised, with lower masks relative to without masks—via increased accuracy, speed, and drift rate. With upper masks, in comparison to without masks, participants were less likely to misjudge surprised expressions as fearful, fearful expressions as surprised, and angry expressions as disgusted, sad expressions as surprised or fearful—via increased accuracy, speed, and evidence accumulation rate.

## Discussion

Across two pre-registered studies collected three and 6 months into the COVID-19 pandemic, we tested whether masks influence how individuals perceive facial expressions of emotion by comparing (i) how accurately individuals judge expressions, (ii) how quickly they make these judgments, and (iii) how rapidly they accumulate evidence for emotion, with and without masks. Participants identified expressions above chance with lower “face masks” and they accumulated evidence towards judging these expressions correctly. However, participants were also less likely—and slower—to identify expressions correctly with lower masks, and they accumulated evidence of emotion more slowly, as compared to without masks. This pattern replicated and intensified in a new sample collected 3 months later. In sum, individuals perceive facial expressions relatively accurately with masks, and yet masks influence communication by slowing the rate at which individuals accumulate evidence of emotion.

By clarifying how masks impact facial emotion communication, these findings could inform interventions to promote mask wearing. Face masks slow the spread of the SARS-CoV-2 virus^51^ and they continue to play a role in the global response to COVID-19^4^, as they likely will for future pandemics and seasonal viruses. However, many individuals have reported not wearing masks due to experiencing difficulties with communication^31,32^. On the one hand, these data show that masks do influence how individuals perceive facial expressions, helping to explain why individuals experience difficulties at times. On the other hand, these data also show that individuals perceive facial expressions well above chance with masks, even without any real-world context, suggesting that concerns over communicating emotion while masked may be overstated. These findings could be used in interventions that acknowledge the challenges individuals may experience, but that also highlight how effectively individuals still communicate emotion when masked^52^.

This research examines emotion perception as a process that unfolds over time and points to settings in which individuals may misperceive masked expressions^53^. Participants were not only less accurate, but also slower at identifying masked expressions, because masks slowed the speed with which they accumulated evidence for emotion. Here, by applying drift–diffusion modeling (DDM)^14,16^^17^, this work extends recent evidence that individuals judge expressions less accurately with face masks^33–41^, by demonstrating that masks also influence the speed of these judgments, and revealing evidence accumulation as the mechanism underlying both of these effects. This suggests that individuals may struggle to perceive masked expressions under time pressure, such as when they quickly pass other people in the grocery store or other brief interactions. Future studies could examine how face masks may impact consumer behavior in these time-sensitive settings.

By showing that masks influence evidence accumulation, this work further predicts that individuals may communicate more effectively by simply taking their time. Face masks influence how individuals judge expressions by slowing the rate at which they accumulate emotion evidence. However, individuals require varying *amounts* of evidence to reach these decisions, depending upon whether they prioritize speed or accuracy when judging emotion^54^. When individuals deprioritize speed, they require more evidence—by increasing the *boundary separation* between evidence thresholds for each response option—and they make fewer errors as a result^13,15^. Here, deprioritizing speed should give individuals time to accumulate more evidence and judge masked expressions more accurately, and thus compensate for masks slowing evidence accumulation. Future investigations could test this model-based prediction by explicitly instructing participants to prioritize either accuracy or speed when judging masked expressions. If successful, this data-driven ‘take your time’ strategy could also be used in interventions to promote mask wearing.

Masks influenced how individuals perceived facial expressions to a greater extent at six versus 3 months into the COVID-19 pandemic. Participants were slower to accumulate evidence of emotion from expressions with lower masks, relative to expressions without masks, at 6 months into the pandemic as compared to an independent sample collected 3 months earlier. However, these groups did not significantly differ in how rapidly they accumulated evidence from expressions with upper masks. This suggests that the effects of masks may have intensified as individuals socially distanced and interacted face-to-face less often. This interpretation is consistent with recent evidence that individuals learned to focus relatively more on the eyes when judging emotion over the first 6 months of the pandemic, and that this adaptation was most pronounced in individuals who were exposed to masked faces more often^55^. However, it is also possible this finding reflects subtle differences between the Radboud Faces Database used in Study 1 and the Racially Diverse Affective Expression (RADIATE) face stimulus set used in Study 2. How else might individuals have adapted to seeing fewer faces in general and fewer lower faces in particular? This question may be particularly important for better understanding potential developmental^56^ and individual^57^ impacts of masks. One possibility is that individuals may have relied more upon context, for example by using more information from individuals’ body postures and their surroundings^6,58,59^.

These data generate fundamental and applied insights into how individuals infer emotion from facial expressions. When directly compared, lower masks interfered more with how participants perceived disgusted, happy, sad, and surprised expressions, whereas upper masks interfered more with how they perceived angry and fearful expressions. Here, upper masks provide an important control by clarifying when lower masks influence emotion judgments by hiding the mouth in particular, versus when they simply conceal half of the face. Upper masks also show how concealing the upper face—with sunglasses for example—may impact emotion communication in daily life. Using both types of masks further reveal which emotion judgments rely more upon information conveyed by either the lower face—such as disgust, happiness, sadness, and surprise—or the upper face—such as anger and fear. These comparisons were made possible by blacking-out either the lower or upper half of each face image. However, while this design choice enhanced experimental control of whether each half of the face was visible^42^, it also limited ecological validity to some extent—for example, by occluding head shape.

In combination with DDM, this design builds on prior research by examining how individuals accumulate evidence for emotion judgments from facial features. Past work found that individuals gaze more towards the eyes of angry, fearful, and sad expressions, versus more towards the mouth of disgusted and happy expressions^25^, and that gazing more towards the eyes increases confusion between anger versus disgust and fear versus surprise, whereas gazing towards the mouth decreases confusion^24,26^. Likewise, we find that individuals accumulate evidence for anger and fear more rapidly from the upper face, whereas they accumulate evidence for disgust, happiness, sadness, and surprise more rapidly from the lower face. These findings suggest that individuals gaze at facial features in ways that generally reflect how they accumulate evidence of emotion from those features. Evidence accumulation further links attention with decision-making as the mechanism by which individuals can make optimal use of facial information when judging emotion^27–29^.

More broadly, this work contributes to theoretical perspectives on communication and social influence by highlighting evidence accumulation as a key mechanism linking social perception and decision-making. Communicative theories propose that individuals produce facial expressions to influence others’ inferences and behavior^60,61^. For instance, “target” individuals generate larger and clearer facial expressions when conveying their experiences to other people^62^. Here, we show that “observer” individuals likewise accumulate evidence for emotion more rapidly from increasingly visible expressions, and that they are more likely—and faster—to perceive these expressions correctly. Evidence accumulation thus provides a core mechanism for facilitating clear communication between targets and observers, and it could help explain recently documented impacts of masks over facial mimicry^63^. Future experiments could explore how evidence accumulation underlies other types of social judgments^64^ and helps individuals connect and cooperate across diverse real-world environments^9,11^, ranging from classrooms^12^ to doctors’ offices^7^ and consumer settings^8^.

As individuals around the world began wearing face masks, they also adapted to how masks influence social interaction. Hospitality staff practiced “smizing”—smiling with the eyes—to convey warm feelings while masked^65^. Just as individuals adapt how they produce expressions behind masks, our findings predict they might also adapt how they perceive masked expressions to improve communication from start to finish.

## Supplementary Information

Supplementary Information.

## Data Availability

Data from all experiments, analysis scripts, plots, and task code are available on GitHub: https://github.com/wcwill/FaceMasksEmotion.
