# Supplementary material for: Face masks influence emotion judgments of facial expressions: a drift–diffusion model
Source: Sci Rep. 2023 May 31;13:8842. doi: 10.1038/s41598-023-35381-4 (PMC10231301; doi:10.1038/s41598-023-35381-4)
Supplement: Supplementary file 1 — Supplementary Information. [file 41598_2023_35381_MOESM1_ESM.docx]

**Supplementary Information**

**

(a) angry faces

(c) fearful faces

(b) disgusted faces

(d) happy faces

(e) sad faces

(f) surprised faces

**

*Supplementary Figure S1*. Mean emotion rating RTs (flipped) by masks and emotion ratings: correct rating RTs vs. false negative error RTs rating (a) angry, (b) disgusted, (c) fearful, (d) happy, (e) sad, and (f) surprised faces, with upper masks vs. no masks (Study 1). Higher mean values reflect faster responses. Error bars depict 95% confidence intervals.

| Supplementary Table S1  *Failing to identify expressions: Upper face mask effects over emotion rating RTs.* | | | | | | |
| --- | --- | --- | --- | --- | --- | --- |
| --- | **Study 1** | | | **Study 2** | | |
| --- | *b* | *CI* | *t* | *b* | *CI* | *t* |
| **All faces** | **---** | **---** | **---** | **---** | **---** | **---** |
| Face mask * rating | -.003 | [-.020, .013] | -.42 | -.009 | [-.027, .010] | -.94 |
| Correct: upper > none | -.006 | [-.013, .002] | -1.53 | -.021 | [-.028, -.014] | -5.42*** |
| Incorrect: upper > none | -.001 | [-.019, .017] | -.14 | -.006 | [-.027, .014] | -.60 |
| **Angry faces** | **---** | **---** | **---** | --- | --- | --- |
| Face mask * rating | -.013 | [-.048, .019] | -.77 | -.014 | [-.064, .035] | -.56 |
| Correct: upper > none | -.033 | [-.052, -.014] | -3.41*** | -.037 | [-.057, -.015] | -3.56*** |
| Incorrect: upper > none | -.018 | [-.050, .010] | -1.20 | -.038 | [-.083, .013] | -1.51 |
| **Disgusted faces** | **---** | **---** | **---** | --- | --- | --- |
| Face mask * rating | -.051 | [-.092, -.012] | -2.57* | .035 | [-.005, .075] | 1.70 |
| Correct: upper > none | -.006 | [-.021, .007] | -.89 | .005 | [-.011, .021] | .56 |
| Incorrect: upper > none | .043 | [-.008, .094] | 1.69 | -.037 | [-.08, .008] | -1.68 |
| **Fearful faces** | **---** | **---** | **---** | --- | --- | --- |
| Face mask * rating | -.026 | [-.065, .015] | -1.31 | -.021 | [-.065, .022] | -.94 |
| Correct: upper > none | -.011 | [-.029, .008] | -1.10 | -.060 | [-.078, -.043] | -6.62** |
| Incorrect: upper > none | .006 | [-.030, .045] | .32 | -.021 | [-.064, .018] | -.99 |
| **Happy faces** | **---** | **---** | **---** | --- | --- | --- |
| Face mask * rating | -.029 | [-.070, .011] | -1.40 | -.038 | [-.083, .007] | -1.61 |
| Correct: upper > none | -.003 | [-.016, .010] | -.42 | -.030 | [-.043, -.015] | -4.08*** |
| Incorrect: upper > none | .026 | [-.028, .083] | .92 | -.006 | [-.056, .040] | -.24 |
| **Sad faces** | **---** | **---** | **---** | --- | --- | --- |
| Face mask * rating | .017 | [-.021, .053] | .90 | -.033 | [-.080, .012] | -1.48 |
| Correct: upper > none | -.009 | [-.023, .006] | -1.25 | -.014 | [-.029, .001] | -1.79 |
| Incorrect: upper > none | -.029 | [-.073, .012] | -1.33 | .032 | [-.022, .088] | 1.11 |
| **Surprised faces** | **---** | **---** | **---** | --- | --- | --- |
| Face mask * rating | -.057 | [-.10, -.017] | -2.64** | -.033 | [-.077, .011] | -1.44 |
| Correct: upper > none | -.025 | [-.040, -.010] | -3.05** | -.013 | [-.029, .002] | -1.62 |
| Incorrect: upper > none | .017 | [-.042, .075] | .58 | .017 | [-.039, .069] | .60 |
| *Note:* * *p* < .05. ** *p* < .01. *** *p* < .001. *CI* = .95. | | | | | | |

**

(a) angry faces

(b) disgusted faces

(c) fearful faces

(f) surprised faces

(e) sad faces

(d) happy faces

**

*Supplementary Figure S2*. Mean emotion rating RTs (flipped) by masks and emotion ratings: correct rating RTs vs. false negative error RTs rating (a) angry, (b) disgusted, (c) fearful, (d) happy, (e) sad, and (f) surprised faces, with lower masks vs. upper masks (Study 1). Higher mean values reflect faster responses. Error bars depict 95% confidence intervals.

| Supplementary Table S2  *Failing to identify expressions: Lower vs. upper face mask effects over emotion rating RTs.* | | | | | | |
| --- | --- | --- | --- | --- | --- | --- |
| --- | **Study 1** | | | **Study 2** | | |
| --- | *b* | *CI* | *t* | *b* | *CI* | *t* |
| **All faces** | **---** | **---** | **---** | **---** | **---** | **---** |
| Face mask * rating | -.022 | [-.036, -.007] | -2.79** | -.002 | [-.018, .016] | -.23 |
| Correct: lower > upper | -.019 | [-.027, -.012] | -4.94*** | -.011 | [-.019, -.003] | -2.62** |
| Incorrect: lower > upper | .003 | [-.011, .018] | .41 | -.008 | [-.023, .009] | -.96 |
| **Angry faces** | **---** | **---** | **---** | **---** | **---** | **---** |
| Face mask * rating | .029 | [-.008, .066] | 1.63 | .060 | [.014, .10] | 2.67** |
| Correct: lower > upper | .033 | [.015, .051] | 3.40*** | .055 | [.038, .073] | 6.03*** |
| Incorrect: lower > upper | .004 | [-.038, .027] | -.23 | .005 | [-.041, .057] | .22 |
| **Disgusted faces** | **---** | **---** | **---** | **---** | **---** | **---** |
| Face mask * rating | -.043 | [-.079, -.006] | -2.25* | -.091 | [-.14, -.048] | -4.28*** |
| Correct: lower > upper | -.057 | [-.075, -.039] | -6.35*** | -.077 | [-.10, -.052] | -6.21*** |
| Incorrect: lower > upper | -.017 | [-.055, .023] | -.86 | .013 | [-.019, .044] | .78 |
| **Fearful faces** | **---** | **---** | **---** | **---** | **---** | **---** |
| Face mask * rating | .029 | [-.008, .063] | 1.61 | .046 | [.0052, .084] | 2.19* |
| Correct: lower > upper | .030 | [.012, .049] | 3.22** | .044 | [.025, .063] | 4.74*** |
| Incorrect: lower > upper | .004 | [-.029, .034] | .23 | -.006 | [-.048, .035] | -.27 |
| **Happy faces** | **---** | **---** | **---** | **---** | **---** | **---** |
| Face mask * rating | -.031 | [-.070, .010] | -1.46 | -.006 | [-.049, .040] | -.24 |
| Correct: lower > upper | -.046 | [-.061, -.032] | -6.28*** | -.040 | [-.057, -.023] | -4.59*** |
| Incorrect: lower > upper | -.013 | [-.066, .038] | -.48 | -.031 | [-.078, .013] | -1.35 |
| **Sad faces** | **---** | **---** | **---** | **---** | **---** | **---** |
| Face mask * rating | -.016 | [-.051, .021] | -.89 | -.007 | [-.048, .035] | -.32 |
| Correct: lower > upper | -.040 | [-.057, -.022] | -4.58*** | -.045 | [-.064, -.025] | -4.63*** |
| Incorrect: lower > upper | -.024 | [-.058, .010] | -1.37 | -.042 | [-.083, -.001] | -1.93 |
| **Surprised faces** | **---** | **---** | **---** | **---** | **---** | **---** |
| Face mask * rating | .001 | [-.043, .038] | -.055 | .005 | [-.033, .043] | .25 |
| Correct: lower > upper | .00 | [-.017, .019] | .098 | -.016 | [-.033, .003] | -1.85 |
| Incorrect: lower > upper | .00 | [-.043, .044] | .015 | -.026 | [-.069, .015] | -1.28 |
| *Note:* * *p* < .05. ** *p* < .01. *** *p* < .001. *CI* = .95. | | | | | | |

**Supplementary Note**

**False Negative Errors**

**Study 2**

***Emotion Ratings***

Participants in Study 2 rated all expressions except disgust correctly on average with lower masks, and all 6 types of expressions with upper masks, largely replicating the results of Study 1 (**Figure 3**). Nonetheless, participants were less accurate rating expressions with lower and upper masks, collapsing across emotions, than without masks (**Table 1, Supplementary Figure S3**). With lower masks, participants rated expressions less accurately for disgust, fear, happiness, sadness, and surprise, but not anger, as compared to without masks. With upper masks, participants rated expressions less accurately for anger and fear, but not for disgust, happiness, sadness, or surprise.

In contrast to Study 1, participants in Study 2 were less accurate rating expressions with lower versus upper masks, collapsing across emotions (**Table 1**). Specifically, participants rated expressions of disgust, happiness, sadness, and surprise less accurately with lower masks versus with upper masks (**Supplementary** **Figure S3**). Conversely, participants were less accurate rating angry expressions with upper masks as compared to lower masks.

(a) angry faces

(b) disgusted faces

(c) fearful faces

(d) happy faces

(e) sad faces

(f) surprised faces

*Supplementary Figure S3*. Mean emotion ratings by masks: correct ratings vs. false negative errors rating (a) angry, (b) disgusted, (c) fearful, (d) happy, (e) sad, and (f) surprised faces (Study 2). Higher mean values reflect increased accuracy and decreased false negative errors. Error bars depict 95% confidence intervals.

***Emotion Rating RTs***

Replicating Study 1, participants in Study 2 were also slower to identify expressions correctly with lower masks (Mdn = 861.39 milliseconds, SD = 562.91), versus without masks (Mdn = 803.38 milliseconds, SD = 516.10), collapsing across emotions (**Figure 3**). In contrast to Study 1, participants were also slower to correctly identify expressions with upper masks (Mdn = 845.04 milliseconds, SD = 538.05) in comparison to without masks, collapsing across emotions (**Supplementary** **Table S1**).

With lower masks, participants were slower to correctly identify expressions of disgust, fear, happiness, sadness, and surprise, but not anger, as compared to without masks, and as indicated by interactions for disgusted and happy expressions (**Table 2, Supplementary Figure S4**). With upper masks, participants were slower to correctly identify angry, fearful, and happy expressions, but not other types of expressions, as compared to without masks.

Replicating Study 1, participants were also slower to correctly identify expressions with lower masks versus with upper masks, collapsing across emotions (**Supplementary** **Table S2**), and in particular for disgusted, happy, and sad expressions, and as shown by an interaction for disgusted expressions. By contrast, participants were slower to correctly identify angry and fearful expressions with upper masks in comparison to lower masks, and as indicated by interactions.

**

(f) surprised faces

(e) sad faces

(b) disgusted faces

(a) angry faces

(c) fearful faces

(d) happy faces

*Supplementary Figure S4*. Mean emotion rating RTs (flipped) by masks and emotion ratings: correct rating RTs vs. false negative error RTs rating (a) angry, (b) disgusted, (c) fearful, (d) happy, (e) sad, and (f) surprised faces, with lower masks vs. no masks (Study 2). Higher mean values reflect faster responses. Error bars depict 95% confidence intervals.

***Emotion Rating Drift Rates***

Replicating the findings of Study 1, participants in Study 2 also accumulated evidence in the direction of correctly rating all 6 types of expressions with lower and upper masks, all P(lower mask < 0) < .0001. Participants also accumulated evidence from expressions with lower and upper masks more slowly, versus without masks, across emotions overall (**Figure 3**). With lower masks, participants accumulated evidence more slowly than without masks for disgust, fear, happiness, sadness, and surprise, all P(lower mask > none) < .0001; but not anger (**Supplementary** **Figure S5**). With upper masks, participants accumulated evidence more slowly than without masks for anger, fear, surprise, all P(upper mask > none) < .0001; disgust, P(upper mask > none) = .034; happiness, P(upper mask > none) = .0004; and sadness, P(upper mask > none) = .0006 (**Supplementary** **Figure S5**).

Again replicating the results of Study 1, participants also accumulated evidence more slowly from expressions with lower masks versus with upper masks, overall across emotions (**Figure 3**). In particular, participants accumulated evidence for disgust, happiness, sadness, and surprise more slowly with lower masks than with upper masks, all P(lower mask > upper mask) < .0001 (**Supplementary** **Figure S5**). Conversely, participants accumulated evidence more slowly for anger and fear from expressions with upper masks than with lower masks, both P(upper mask > lower mask) < .0001.

**

(a) angry faces

(b) disgusted faces

(c) fearful faces

(d) happy faces

(e) sad faces

(f) surprised faces

**

*Supplementary Figure S5*. Drift rate coefficients by masks: drift rate towards correct judgments vs. false negative errors rating (a) angry, (b) disgusted, (c) fearful, (d) happy, (e) sad, and (f) surprised faces (Study 2). Higher coefficient values reflect increased drift rate towards correct judgments and decreased drift rate towards false negative errors. Error bars depict 95% credible intervals.

***Emotion Rating Boundary Separations***

Replicating Study 1, Study 2 participants did not require a significantly different amount of evidence for identifying expressions with lower masks, relative to without masks. However, unlike Study 1, participants in Study 2 showed no significant difference in how much evidence they required for identifying expressions with upper masks compared to without masks, collapsing across emotions.

With upper masks, participants required more evidence for identifying happiness with upper masks (*b* = 1.53, 95% CI [1.46, 1.60]) than without masks (*b* = 1.42, 95% CI [1.34, 1.50]), P(upper mask < none) = .0002; but did not show a significant difference in how much evidence they required for identifying anger, disgust, fear, sadness, and surprise. With lower masks, participants required more evidence for identifying happiness with lower masks (*b* = 1.54, 95% CI [1.48, 1.61]) than without masks (*b* = 1.42, 95% CI [1.34, 1.50]), P(lower mask < none) < .0001); but showed no significant difference for anger, disgust, fear, sadness, and surprise.

Unlike in Study 1, participants in Study 2 did not significantly differ in the amount of evidence they required when identifying expressions with lower masks versus upper masks, collapsing across emotions. This pattern was consistent across anger, disgust, happiness, sadness, and surprise. However, they did require less evidence for identifying fear with lower masks (*b* = 1.54, 95% CI [1.47, 1.60]) than with upper masks (*b* = 1.61, 95% CI [1.54, 1.68]), P(lower mask > upper mask) = .016.

**Supplementary Note**

**False Positive Errors**

**Study 1**

***Misidentifying Expressions with Lower Masks***

**Anger Judgments.** Participants were less likely to judge expressions of disgust, happiness, and surprise, as not angry with lower masks as compared to without masks (**Supplementary** **Table S3**), and slower to judge surprised expressions as not angry, *b* = -.030, *t* = -3.95, *p* < .001, 95% CI [-.046, -.015]. They also accumulated evidence that surprised expressions were not angry more slowly with lower masks (*b* = 1.53, 95% CI [1.43, 1.63]) versus without masks (*b* = 1.77, 95% CI [1.54, 2.01]), P(lower mask > none) < .0001.

**Disgust Judgments.** Participants judged angry expressions as not disgusted less often with lower masks, in comparison to without masks (**Supplementary** **Table S4**). However, they were also faster to judge angry expressions as not disgusted with lower masks versus without masks, *b* = .021, *t* = 2.21, *p* = .027, 95% CI [.001, .038].

Conversely, participants judged fearful expressions as not disgusted more often with lower masks versus without masks—and more quickly, *b* = .045, *t* = 4.89, *p* < .001, 95% CI [.028, .063], and they were also faster to accumulate evidence that fearful expressions were not disgusted with lower masks (*b* = .85, 95% CI [.75, .95]) than without masks (*b* = .17, 95% CI [.004, .34]), P(lower mask < none) < .0001).

**Fear Judgments.** Participants judged angry and sad expressions as not fearful less often with lower masks than without masks (**Supplementary** **Table S5**), but they showed no significant differences in the speed of these ratings.

**Happiness Judgments.** Participants were less likely to judge angry, disgusted, sad, and surprised expressions as not happy with lower masks relative to without masks (**Supplementary** **Table S6**)—and slower for expressions of anger (*b* = -.017, *t* = -2.42, *p* = .016, 95% CI [-.031, -.003]), disgust (*b* = -.028, *t* = -4.02, *p* < .001, 95% CI [-.042, -.014]), sadness (*b* = -.033, *t* = -5.31, *p* < .001, 95% CI [-.045, -.022]), and surprise (*b* = -.018, *t* = -2.45, *p* = .014, 95% CI [-.034, -.003]). In addition, they also accumulated evidence that expressions were not happy more slowly with lower masks versus without masks, for expressions of anger (lower mask: *b* = 2.36, 95% CI [2.24, 2.47]; none: *b* = 2.60, 95% CI [2.31, 2.88]), disgust (lower mask: *b* = 1.91, 95% CI [1.78, 2.03]; none: *b* = 2.34, 95% CI [2.10, 2.59]), sadness (lower mask: *b* = 2.08, 95% CI [1.96, 2.19]; none: *b* = 2.55, 95% CI [2.30, 2.83]), and surprise (lower mask: *b* = 1.61, 95% CI [1.49, 1.73]; none: *b* = 1.90, 95% CI [1.68, 2.12]), all P(lower mask > none) < .0001.

**Sadness Judgments.** Participants judged happy expressions as not sad less frequently with lower masks as compared to without masks (**Supplementary** **Table S7**), but they did not significantly differ in their speed making these ratings.

In contrast, participants judged angry and fearful expressions as not sad more frequently with lower masks versus without masks—and they were faster to judge fearful expressions as not sad, *b* = .030, *t* = 3.28, *p* = .0011, 95% CI [.013, .048]. In addition, participants accumulated evidence that fearful expressions were not sad more quickly with lower masks (*b* = .99, 95% CI [.88, 1.09]) than without masks (*b* = .65, 95% CI [.48, .84]), P(lower mask < none) < .0001.

**Surprise Judgments.** Participants were less likely to judge fearful and sad expressions as not surprised with lower masks in comparison to without masks (**Supplementary** **Table S8**)—and slower to judge sad expressions as not surprised, *b* = -.023, *t* = -2.88, *p* = .0041, 95% CI [-.039, -.008]. Participants also accumulated evidence that sad expressions were not surprised more slowly with lower masks (*b* = 1.47, 95% CI [1.37, 1.58]) than without masks (*b* = 1.79, 95% CI [1.58, 2.01]), P(lower mask > none) < .0001.

***Misidentifying Expressions with Upper Masks***

**Anger Judgments.** Participants rated sad expressions as not angry less often with upper masks, as compared to without masks, but they showed no significant differences in how quickly they rated sad expressions.

By contrast, participants rated disgusted expressions as not angry more often with upper masks than without masks—and more quickly, *b* = .051, *t* = 4.61, *p* < .001, 95% CI [.027, .072]—and they were faster to accumulate evidence that disgusted expressions were not angry with upper masks (*b* = .13, 95% CI [.036, .23]) versus without masks (*b* = -.17, 95% CI [-.31, -.028]), P(upper mask < none) < .0001.

**Disgust Judgments.** Participants were less likely to rate fearful expressions as not disgusted with upper masks, versus without masks, but they did not significantly differ in their speed making these ratings.

Conversely, participants were more likely to rate angry expressions as not disgusted with upper masks than without masks—and faster, *b* = .052, *t* = 5.90, *p* < .001, 95% CI [.034, .069]— and they accumulated evidence more quickly that angry expressions were not disgusted with upper masks (*b* = .89, 95% CI [.78, .99]) as compared to without masks (*b* = .51, 95% CI [.32, .70]), P(upper mask < none) < .0001.

**Fear Judgments.** Participants rated angry, sad, and surprised expressions as not fearful more frequently with upper masks relative to without masks—and more quickly for expressions of anger (*b* = .039, *t* = 4.87, *p* < .001, 95% CI [.023, .056]), sadness (*b* = .025, *t* = 2.86, *p* = .0043, 95% CI [.008, .044]), and surprise (*b* = .039, *t* = 2.81, *p* = .0051, 95% CI [.012, .064]). They were also faster to accumulate evidence that expressions were not fearful with upper masks versus without masks, for expressions of anger (upper mask: *b* = 1.30, 95% CI [1.19, 1.40]; none: *b* = 1.04, 95% CI [.83, 1.25]), sadness (upper mask: *b* = .93, 95% CI [.83, 1.04]; none: *b* = .70, 95% CI [.51, .89]), and surprise (upper mask: *b* = -.015, 95% CI [-.11, .078]; none: *b* = -.33, 95% CI [-.49, -.17]), all P(upper mask < none) < .0001.

**Happiness Judgments.** Participants rated angry expressions as not happy less often with upper masks in comparison to without masks, but they showed no significant differences in how quickly they made these ratings.

**Sadness Judgments.** Participants were less likely to rate angry and fearful expressions as not sad with upper masks than without masks, but they did not significantly differ in their speed making these ratings.

**Surprise Judgments.** Participants rated angry and fearful expressions as not surprised more often with upper masks versus without masks—and they rated fearful expressions as not surprised more quickly, *b* = .068, *t* = 6.62, *p* < .001, 95% CI [.047, .086]. In addition, participants were also faster to accumulate evidence that fearful expressions were not surprised with upper masks (*b* = .92, 95% CI [.82, 1.02]) than without masks (*b* = .018, 95% CI [-.11, .15]), P(upper mask < none) < .0001.

***Misidentifying Expressions with Lower versus Upper Masks***

**Anger Judgments.** Participants were less likely to judge disgusted and happy expressions as not angry with lower masks than with upper masks—and slower for both disgusted (*b* = -.035, *t* = -2.56, *p* = .011, 95% CI [-.059, -.006]) and happy (*b* = -.024, *t* = -3.31, *p* < .001, 95% CI [-.038, -.010]) expressions. They also accumulated evidence more slowly that expressions were not angry with lower versus upper masks, for expressions of disgust (lower mask: *b* = -.80, 95% CI [-.90, -.71]; upper mask: *b* = .13, 95% CI [.036, .23]) and happiness (lower mask: *b* = 2.20, 95% CI [2.08, 2.31]; upper mask: *b* = 2.49, 95% CI [2.38, 2.60]), both P(lower mask > upper mask) < .0001.

By contrast, participants were less likely to judge fearful expressions as not angry with upper versus lower masks, but they did not show significant differences in their speed making these ratings.

**Disgust Judgments.** Participants judged angry expressions as not disgusted less often with lower masks relative to with upper masks—and more slowly, *b* = -.035, *t* = -4.25, *p* < .001, 95% CI [-.052, -.019]—and they were slower to accumulate evidence that angry expressions were not disgusted with lower masks (*b* = .43, 95% CI [.34, .53]) as compared to with upper masks (*b* = .89, 95% CI [.78, .99]), P(lower mask > upper mask) < .0001.

In contrast, participants judged fearful and surprised expressions as not disgusted less often with upper masks than with lower masks—and they were slower for fearful expressions, *b* = .033, *t* = 3.06, *p* = .0022, 95% CI [.011, .054]. Consistently, participants also accumulated evidence more slowly that fearful expressions were not disgusted with upper masks (*b* = -.14, 95% CI [-.23, -.044]) in comparison to with lower masks (*b* = .85, 95% CI [.75, .95]), P(upper mask > lower mask) < .0001.

**Fear Judgments.** Participants judged angry, happy, sad, and surprised expressions as not fearful less often with lower masks as compared to with upper masks—and they were slower for expressions of anger (*b* = -.043, *t* = -5.09, *p* < .001, 95% CI [-.060, -.027]), happiness (*b* = -.018, *t* = 2.36, *p* = .019, 95% CI [-.033, -.003]), and sadness (*b* = -.027, *t* = -2.98, *p* = .0029, 95% CI [-.045, -.009]). They also accumulated evidence more slowly that expressions were not fearful with lower versus upper masks, for angry (lower mask: *b* = .89, 95% CI [.79, .99]; upper mask: *b* = 1.30, 95% CI [1.19, 1.40]), happy (lower mask: *b* = 2.02, 95% CI [1.91, 2.13]; upper mask: *b* = 2.25, 95% CI [2.14, 2.37]), and sad (lower mask: *b* = .44, 95% CI [.34, .54]; upper mask: *b* = .93, 95% CI [.83, 1.04]) expressions, all P(lower mask > upper mask) < .0001.

**Happiness Judgments.** Participants were less likely to judge disgusted and surprised expressions as not happy with lower masks than with upper masks—and slower for both disgusted (*b* = -.021, *t* = -3.21, *p* = .0014, 95% CI [-.035, -.009]) and surprised (*b* = -.025, *t* = -3.67, *p* < .001, 95% CI [-.038, -.011]) expressions. Likewise, they also accumulated evidence more slowly that expressions were not happy with lower versus upper masks, for expressions of disgust (lower mask: *b* = 1.91, 95% CI [1.78, 2.03]; upper mask: *b* = 2.19, 95% CI [2.07, 2.32]) and surprise (lower mask: *b* = 1.61, 95% CI [1.49, 1.73]; upper mask: *b* = 1.95, 95% CI [1.83, 2.07]), both P(lower mask > upper mask) < .0001.

**Sadness Judgments.** Participants were less likely to judge happy expressions as not sad with lower masks in comparison to with upper masks—and slower, *b* = -.016, *t* = -2.22, *p* = .027, 95% CI [-.032, -.002]—and they accumulated evidence more slowly that happy expressions were not sad with lower masks (*b* = 2.13, 95% CI [2.01, 2.25]) than with upper masks (*b* **=** 2.38, 95% CI [2.26, 2.50]), P(lower mask > upper mask) < .0001.

Conversely, participants were less likely to judge angry and fearful expressions as not sad with upper masks as compared to with lower masks—and slower for both angry (*b* = .031, *t* = 2.56, *p* = .011, 95% CI [.006, .053]) and fearful (*b* = .031, *t* = 3.70, *p* < .001, 95% CI [.015, .049]) expressions. They also accumulated evidence more slowly that expressions were not sad with upper versus lower masks, for expressions of anger (upper mask: *b* = .017, 95% CI [-.080, .11]; lower mask: *b* = .56, 95% CI [.46, .65]) and fear (upper mask: *b* = .40, 95% CI [.29, .50]; lower mask: *b* = .99, 95% CI [.88, 1.09]), both P(upper mask > lower mask) < .0001.

**Surprise Judgments.** Participants judged all five other types of expressions as not surprised less often with lower masks than with upper masks—and more slowly for expressions of anger (*b* = -.021, *t* = -2.77, *p* = .0057, 95% CI [-.036, -.007]), disgust (*b* = -.016, *t* = -2.17, *p* = .030, 95% CI [-.030, -.002]), fear (*b* = -.033, *t* = -2.82, *p* = .0049, 95% CI [-.056, -.010]), happiness (*b* = -.042, *t* = -4.76, *p* < .001, 95% CI [-.059, -.022]), and sadness (*b* = -.058, *t* = -7.33, *p* < .001, 95% CI [-.075, -.044]). In addition, they were slower to accumulate evidence that expressions were not surprised with lower versus upper masks, for angry (lower mask: *b* = 1.79, 95% CI [1.68, 1.90]; upper mask: *b* = 2.06, 95% CI [1.95, 2.16]), disgusted (lower mask: *b* = 1.64, 95% CI [1.53, 1.75]; upper mask: *b* = 1.84, 95% CI [1.73, 1.94]), fearful (lower mask: *b* = -.69, 95% CI [-.79, -.59]; upper mask: *b* = .92, 95% CI [.82, .102]), happy (lower mask: *b* = 1.61, 95% CI [1.50, 1.71]; upper mask: *b* = 1.91, 95% CI [1.80, 2.01]), and sad (lower mask: *b* = 1.47, 95% CI [1.37, 1.58]; upper mask: *b* = 2.02, 95% CI [1.90, 2.13]) expressions, all P(lower mask > upper mask) < .0001.

**Study 2**

***Misidentifying Expressions with Lower Masks***

**Anger Judgments.** Participants rated disgusted, fearful, and surprised expressions as not angry less frequently with lower masks relative to without masks (**Supplementary** **Table S3**)—and more slowly for surprised expressions, *b* = -.027, *t* = -3.02, *p* = .0026, 95% CI [-.044, -.010]. In addition, participants were also slower to accumulate evidence that surprised expressions were not angry with lower masks (*b* = 2.16, 95% CI [2.02, 2.32]) than without masks (*b* = 2.58, 95% CI [2.33, 2.83]), P(lower mask > none) < .0001.

**Disgust Judgments.** Participants rated fearful expressions as not disgusted less often with lower masks than without masks (**Supplementary** **Table S4**). However, they were also faster to rate fearful expressions as not disgusted, with lower masks versus without masks, *b* = .048, *t* = 4.30, *p* < .001, 95% CI [.026, .071].

**Fear Judgments.** Participants were less likely to rate angry expressions as not fearful with lower masks in comparison to without masks (**Supplementary** **Table S5**), but they did not significantly differ in their speed making these ratings.

**Happiness Judgments.** Participants rated sad expressions as not happy less frequently with lower masks relative to without masks (**Supplementary** **Table S6**)—and more slowly, *b* = -.031, *t* = -3.57, *p* < .001, 95% CI [-.049, -.014], and they were slower to accumulate evidence that sad expressions were not happy with lower masks (*b* = 2.28, 95% CI [2.11, 2.45]) than without masks (*b* = 2.73, 95% CI [2.44, 3.03]), P(lower mask > none) < .0001.

**Sadness Judgments.** Participants were less likely to rate expressions of disgust, happiness, and surprise, as not sad with lower masks as compared to without masks (**Supplementary** **Table S7**)—and slower for both happy (*b =* -.027, *t =* -3.40, *p <* .001, 95% CI [-.043, -.011]) and surprised (*b =* -.031, *t =* -3.28, *p =* .0011, 95% CI [-.049, -.012]) expressions. Consistently, they also accumulated evidence more slowly that these expressions were not sad with lower masks than without masks, for expressions of happiness (lower mask: *b* = 2.31, 95% CI [2.13, 2.48]; none: *b* = 2.79, 95% CI [2.56, 3.03]) and surprise (lower mask: *b* = 1.80, 95% CI [1.65, 1.94]; none: *b* = 2.15, 95% CI [1.92, 2.38]), both P(lower mask > none) < .0001.

By contrast, participants were more likely to rate angry expressions as not sad with lower masks than without masks—and faster, *b =* .049, *t =* 4.93, *p <* .001, 95% CI [.030, .068], and they accumulated evidence more quickly that angry expressions were not sad with lower masks (*b =* 1.15, 95% CI [1.00, 1.29]) than without masks (*b =* 0.65, 95% CI [.48, .82]), P(lower mask < none) < .0001.

**Surprise Judgments.** Participants rated angry expressions as not surprised less often with lower masks as compared to without masks (**Supplementary** **Table S8**)—and less quickly, *b =* -.027, *t =* -3.37, *p <* .001, 95% CI [-.043, -.012], and they were slower to accumulate evidence that angry expressions were not surprised with lower masks (*b =* 1.80, 95% CI [1.64 1.96]) than without masks (*b =* 2.27, 95% CI [2.03, 2.50], P(lower mask > none) < .0001.

Conversely, participants rated disgusted expressions as not surprised more often with lower masks than without masks—and faster, *b =* .055, *t =* 5.29, *p <* .001, 95% CI [.034, .076], and they accumulated evidence more quickly that disgusted expressions were not surprised with lower masks (*b =* 1.37, 95% CI [1.22, 1.52]) versus without masks (*b =* .77, 95% CI [.59, .96]), P(lower mask < none) < .0001.

***Misidentifying Expressions with Upper Masks***

**Anger Judgments.** There were no significant differences in how often participants misidentified expressions as angry with upper masks relative to without masks.

**Disgust Judgments.** Participants judged fearful expressions as not disgusted less often with upper masks, in comparison to without masks, but they did not significantly differ in their speed making these ratings.

In contrast, participants judged angry expressions as not disgusted more often with upper masks relative to without masks—and more quickly, *b =* .043, *t =* 4.81, *p <* .001, 95% CI [.027, .061], and they were faster to accumulate evidence that angry expressions were not disgusted with upper masks (*b =* 1.08, 95% CI [.93, 1.22]) than without masks (*b* = .67, 95% CI [.50, .83]), P(upper mask < none) < .0001.

**Fear Judgments.** Participants were more likely to judge sad and surprised expressions as not fearful with upper masks than without masks—and faster for both sad (*b =* .026, *t =* 3.05, *p* = .0024, 95% CI [.009, .044]) and surprised (*b* = .038, *t =* 2.40, *p* = .017, 95% CI [.004, .069]) expressions. Likewise, they also accumulated evidence more quickly that these expressions were not fearful with upper masks versus without masks, for expressions of sadness (upper mask: *b =* 1.49, 95% CI [1.34, 1.64]; none: *b =* 1.08, 95% CI [.92, 1.26]) and surprise (upper mask: *b =* -.14, 95% CI [-.28, -.009]; none: *b* = -.54, 95% CI [-.68, -.40]), both P(upper mask < none) < .0001.

**Happiness Judgments.** We did not detect significant differences in how frequently participants misidentified expressions as happy with upper masks compared to without masks.

**Sadness Judgments.** Participants judged angry expressions as not sad less frequently with upper masks versus without masks, but they showed no significant differences in how quickly they made these ratings.

**Surprise Judgments.** Participants judged fearful and sad expressions as not surprised more often with upper masks than without masks—and more quickly for both fearful (*b* = .040, *t =* 2.54, *p* = .012, 95% CI [.010, .071]) and sad (*b* = .017, *t =* 2.15, *p* = .032, 95% CI [.002, .032]) expressions. They were also faster to accumulate evidence that these expressions were not surprised with upper masks versus without masks, for expressions of fear (upper mask: *b* = -.070, 95% CI [-.20, .064]; none: *b* = -.51, 95% CI [-.65, -.36]; P(upper mask < none) < .0001) and sadness (upper mask: *b =* 2.69, 95% CI [2.52, 2.86]; none: *b =* 2.38, 95% CI [2.12, 2.62]; P(upper mask < none) = .0002).

***Misidentifying Expressions with Lower versus Upper Masks***

**Anger Judgments.** Participants were less likely to rate disgusted, happy, and surprised expressions as not angry with lower masks relative to with upper masks—and slower for disgusted (*b* = -.044, *t =* -3.21, *p* < .01, 95% CI [-.073, -.019]), happy (*b* = -.020, *t =* -2.84, *p* = .0046, 95% CI [-.034, -.006]), and surprised (*b* = -.021, *t =* -2.33, *p* = .020, 95% CI [-.039, -.002]) expressions. In addition, they accumulated evidence more slowly that these expressions were not angry with lower versus upper masks, for expressions of disgust (lower mask: *b* = -.59, 95% CI [-.73, -.44]; upper mask: *b* = .84, 95% CI [.70, .98]), happiness (lower mask: *b* = 2.56, 95% CI [2.39, 2.73]; upper mask: *b* = 2.91, 95% CI [2.74, 3.09]), and surprise (lower mask: *b* = 2.17, 95% CI [2.02, 2.32]; upper mask: *b* = 2.53, 95% CI [2.38, 2.68]), all P(lower mask > upper mask) < .0001.

**Disgust Judgments.** Participants were less likely to rate angry expressions as not disgusted with lower masks as compared to with upper masks—and slower, *b* = -.033, *t =* -3.57, *p* < .001, 95% CI [-.049, -.016], and they accumulated evidence that angry expressions were not disgusted more slowly with lower (*b =* .65, 95% CI [.51, .79]) versus upper masks (*b =* 1.08, 95% CI [.93, 1.22]), P(lower mask > upper mask) < .0001.

Conversely, participants were less likely to rate fearful expressions as not disgusted with upper masks in comparison to with lower masks—and slower, *b* = .044, *t =* 3.96, *p* < .001, 95% CI [.023, .065], and they accumulated evidence that fearful expressions were not disgusted more slowly with upper (*b =* .11, 95% CI [-.025, .24]) versus lower masks (*b =* .87, 95% CI [.73, 1.01]), P(upper mask > lower mask) < .0001.

**Fear Judgments.** Participants rated angry and sad expressions as not fearful less frequently with lower masks than with upper masks—and more slowly for both angry (*b* = -.033, *t =* -4.12, *p* < .001, 95% CI [-.049, -.017]) and sad (*b* = -.022, *t =* -2.43, *p* = .015, 95% CI [-.038, -.004]) expressions. Consistently, they were also slower to accumulate evidence that these expressions were not fearful with lower versus upper masks, for expressions of anger (lower mask: *b =* 1.57, 95% CI [1.41, 1.71]; upper mask: *b =* 2.18, 95% CI [2.03, 2.33]) and sadness (lower mask: *b =* .67, 95% CI [.52, .82]; upper mask: *b =* 1.49, 95% CI [1.34, 1.64]), both P(lower mask > upper mask) < .0001.

**Happiness Judgments.** Participants rated sad expressions as not happy less often with lower masks in comparison to with upper masks—and more slowly, *b =* -.029, *t* = -3.41, *p* < .001, 95% CI [-.045, -.011], and they were slower to accumulate evidence that sad expressions were not happy with lower masks (*b* = 2.28, 95% CI [2.11, 2.45]) versus upper masks (*b* = 2.75, 95% CI [2.57, 2.94]), P(lower mask > upper mask) < .0001.

**Sadness Judgments.** Participants were less likely to rate disgusted, happy, and surprised expressions as not sad with lower masks relative to with upper masks—and slower for disgusted (*b =* -.020, *t* = -2.27, *p* = .023, 95% CI [-.037, -.002]), happy (*b =* -.030, *t* = -3.83, *p* < .001, 95% CI [-.046, -.014]), and surprised (*b =* -.031, *t* = -3.30, *p* = .0010, 95% CI [-.050, -.012]) expressions. They also accumulated evidence that these expressions were not sad more slowly with lower versus upper masks, for expressions of disgust (lower mask: *b* = 1.27, 95% CI [1.13 1.42]; upper mask: *b* = 1.79, 95% CI [1.63, 1.94]), happiness (lower mask: *b* = 2.31, 95% CI [2.13, 2.48]; upper mask: *b* = 2.85, 95% CI [2.68, 3.01]), and surprise (lower mask: *b* = 1.80, 95% CI [1.65, 1.94]; upper mask: *b* = 2.23, 95% CI [2.09, 2.38]), P(lower mask > upper mask) < .0001.

By contrast, participants were less likely to rate angry expressions as not sad with upper masks as compared to with lower masks—and slower, *b =* .055, *t* = 5.26, *p* < .001, 95% CI [.033, .074], and they accumulated evidence that angry expressions were not sad more slowly with upper (*b =* .28, 95% CI [.14, .42]) versus lower masks (*b =* 1.15, 95% CI [1.00, 1.29]), P(upper mask > lower mask) < .0001.

**Surprise Judgments.** Participants rated angry, fearful, and sad expressions as not surprised less frequently with lower masks than with upper masks—and more slowly for angry (*b =* -.043, *t* = -5.89, *p* < .001, 95% CI [-.057, -.028]) and sad (*b =* -.042, *t* = -5.20, *p* < .001, 95% CI [-.058, -.026]) expressions. In addition, they were slower to accumulate evidence that these expressions were not surprised with lower versus upper masks, for expressions of anger (lower mask: *b =* 1.80, 95% CI [1.64, 1.96]; upper mask: *b* = 2.65, 95% CI [2.48, 2.82]) and sadness (lower mask: *b =* 1.94, 95% CI [1.77, 2.10]; upper mask: *b =* 2.69, 95% CI [2.52, 2.86], P(lower mask > upper mask) < .0001.

In contrast, participants rated disgusted expressions as not surprised less often with upper masks relative to with lower masks—and more slowly, *b =* .031, *t* = 3.56, *p* < .001, 95% CI [.014, .049], and they were also slower to accumulate evidence that disgusted expressions were not surprised with upper (*b =* .85, 95% CI [.71, 1.00]) versus lower masks (*b =* 1.37, 95% CI [1.22, 1.52]), P(upper mask > lower mask) < .0001.

| Supplementary Table S3  *Misidentifying expressions: Face mask effects over anger ratings.* | | | | | | |
| --- | --- | --- | --- | --- | --- | --- |
| --- | **Study 1** | | | **Study 2** | | |
| --- | *b* | *CI* | *z* | *b* | *CI* | *z* |
| **Disgusted faces** | **---** | **---** | **---** | **---** | **---** | **---** |
| Lower mask > none | -1.41 | [-1.73, -1.09] | -8.35*** | -2.41 | [-3.27, -1.58] | -5.62*** |
| Lower mask > upper | -1.93 | [-2.25, -1.63] | -11.79*** | -2.58 | [-3.32, -1.74] | -6.31*** |
| Upper mask > none | .56 | [.22, .88] | 3.39*** | .37 | [-.50, 1.22] | .86 |
| **Fearful faces** | **---** | **---** | **---** | **---** | **---** | **---** |
| Lower mask > none | .24 | [-.14, 51] | 1.73 | -.82 | [-1.44, -.14] | -2.49* |
| Lower mask > upper | .35 | [.086, .65] | 2.56* | -.34 | [-.96, .33] | -1.01 |
| Upper mask > none | -.13 | [-.39, .11] | -1.00 | -.45 | [-1.09, .27] | -1.37 |
| **Happy faces** | **---** | **---** | **---** | **---** | **---** | **---** |
| Lower mask > none | -.50 | [-1.07, -.054] | -2.08* | -.78 | [-2.08, .13] | -1.61 |
| Lower mask > upper | -.33 | [-.64, -.018] | -2.07* | -.97 | [-2.89, -.095] | -2.69** |
| Upper mask > none | .077 | [-.47, .66] | .30 | .10 | [-1.06, 1.18] | .21 |
| **Sad faces** | **---** | **---** | **---** | **---** | **---** | **---** |
| Lower mask > none | -.20 | [-.57, .14] | -1.10 | -.39 | [-1.17, .45] | -.93 |
| Lower mask > upper | .18 | [-.066, .42] | 1.44 | -.056 | [-.80, .77] | -.14 |
| Upper mask > none | -.48 | [-.80, -.10] | -2.60** | -.34 | [-1.15, .52] | -.81 |
| **Surprised faces** | **---** | **---** | **---** | **---** | **---** | **---** |
| Lower mask > none | -.33 | [-.64, -.028] | -2.08* | -1.04 | [-2.01, -.48] | -3.35*** |
| Lower mask > upper | -.17 | [-.56, .21] | -1.02 | -1.32 | [-2.44, -.87] | -4.02*** |
| Upper mask > none | -.15 | [-.49, .20] | -.94 | .26 | [-.46, 1.03] | .78 |
| *Note:* * *p* < .05. ** *p* < .01. *** *p* < .001. *CI* = .95. | | | | | | |

| Supplementary Table S4  *Misidentifying expressions: Face mask effects over disgust ratings.* | | | | | | |
| --- | --- | --- | --- | --- | --- | --- |
| --- | **Study 1** | | | **Study 2** | | |
| --- | *b* | *CI* | *z* | *b* | *CI* | *z* |
| **Angry faces** | **---** | **---** | **---** | **---** | **---** | **---** |
| Lower mask > none | -.25 | [-.51, -.022] | -2.00* | -.075 | [-.73, .58] | -.23 |
| Lower mask > upper | -.90 | [-1.17, -.63] | -6.97*** | -.79 | [-1.49, -.074] | -2.27* |
| Upper mask > none | .69 | [.43, .94] | 5.29*** | .75 | [.075, 1.39] | 2.21* |
| **Fearful faces** | **---** | **---** | **---** | **---** | **---** | **---** |
| Lower mask > none | 1.30 | [1.00, 1.60] | 8.36*** | .60 | [.055, 1.10] | 2.27* |
| Lower mask > upper | 1.78 | [1.48, 2.09] | 11.32*** | 1.31 | [.79, 1.82] | 5.09*** |
| Upper mask > none | -.55 | [-.83, -.27] | -3.70*** | -.76 | [-1.32, -.21] | -2.93** |
| **Happy faces** | **---** | **---** | **---** | **---** | **---** | **---** |
| Lower mask > none | -.25 | [-.69, .20] | -1.32 | .16 | [-.79, 1.23] | .36 |
| Lower mask > upper | .027 | [-.47, .51] | .22 | -.53 | [-1.99, .43] | -1.14 |
| Upper mask > none | -.26 | [-.71, .18] | -1.39 | .74 | [-.13, 1.91] | 1.65 |
| **Sad faces** | **---** | **---** | **---** | **---** | **---** | **---** |
| Lower mask > none | -.006 | [-.25, .24] | -.042 | -.31 | [-.96, .42] | -.90 |
| Lower mask > upper | -.14 | [-.38, .13] | -1.05 | -.16 | [-.85, .55] | -.43 |
| Upper mask > none | .13 | [-.13, .38] | 1.01 | -.14 | [-.83, .53] | -.42 |
| **Surprised faces** | **---** | **---** | **---** | **---** | **---** | **---** |
| Lower mask > none | .26 | [-.030, .54] | 1.78 | .17 | [-.28, .65] | .71 |
| Lower mask > upper | .35 | [.084, .66] | 2.50* | -.026 | [-.48, .43] | -.11 |
| Upper mask > none | -.096 | [-.38, .19] | -.67 | .19 | [-.22, .68] | .81 |
| *Note:* * *p* < .05. ** *p* < .01. *** *p* < .001. *CI* = .95. | | | | | | |

| Supplementary Table S5  *Misidentifying expressions: Face mask effects over fear ratings.* | | | | | | |
| --- | --- | --- | --- | --- | --- | --- |
| --- | **Study 1** | | | **Study 2** | | |
| --- | *b* | *CI* | *z* | *b* | *CI* | *z* |
| **Angry faces** | **---** | **---** | **---** | **---** | **---** | **---** |
| Lower mask > none | -.36 | [-.65, -.083] | -2.45* | -.58 | [-1.07, -.063] | -2.23* |
| Lower mask > upper | -.63 | [-.90, -.32] | -4.18*** | -1.01 | [-1.55, -.46] | -3.66*** |
| Upper mask > none | .31 | [.026, .63] | 2.04* | .47 | [-.084, .99] | 1.67 |
| **Disgusted faces** | **---** | **---** | **---** | **---** | **---** | **---** |
| Lower mask > none | .21 | [-.087, .89] | 1.44 | .074 | [-.53, .67] | .24 |
| Lower mask > upper | .088 | [-.19, .37] | .61 | .084 | [-.53, .75] | .26 |
| Upper mask > none | .11 | [-.19, .39] | .79 | -.034 | [-.61, .55] | -.11 |
| **Happy faces** | **---** | **---** | **---** | **---** | **---** | **---** |
| Lower mask > none | -.26 | [-.75, .17] | -1.38 | -.68 | [-1.77, .087] | -1.65 |
| Lower mask > upper | -.43 | [-.99, -.010] | -2.17* | -.48 | [-1.41, .15] | -1.42 |
| Upper mask > none | .15 | [-.30, .62] | .76 | -.23 | [-1.22, .60] | -.55 |
| **Sad faces** | **---** | **---** | **---** | **---** | **---** | **---** |
| Lower mask > none | -.53 | [-.79, -.27] | -3.97*** | -.69 | [-1.45, .012] | -1.85 |
| Lower mask > upper | -.88 | [-1.11, -.59] | -6.33*** | -1.36 | [-2.14, -.58] | -3.47*** |
| Upper mask > none | .37 | [.098, .64] | 2.69** | .79 | [.008, 1.52] | 2.05* |
| **Surprised faces** | **---** | **---** | **---** | **---** | **---** | **---** |
| Lower mask > none | -.027 | [-.31, .25] | -.19 | .29 | [-.33, .96] | .87 |
| Lower mask > upper | -.68 | [-.98, -.39] | -4.58*** | -.44 | [-1.02, .17] | -1.43 |
| Upper mask > none | .67 | [.38, .97] | 4.58*** | .75 | [.091, 1.38] | 2.28* |
| *Note:* * *p* < .05. ** *p* < .01. *** *p* < .001. *CI* = .95. | | | | | | |

| Supplementary Table S6  *Misidentifying expressions: Face mask effects over happiness ratings.* | | | | | | |
| --- | --- | --- | --- | --- | --- | --- |
| --- | **Study 1** | | | **Study 2** | | |
| --- | *b* | *CI* | *z* | *b* | *CI* | *z* |
| **Angry faces** | **---** | **---** | **---** | **---** | **---** | **---** |
| Lower mask > none | -.72 | [-1.35, -.23] | -2.62** | -.075 | [-1.17, .97] | -.15 |
| Lower mask > upper | .11 | [-.48, .75] | .41 | -.37 | [-1.96, .84] | -.59 |
| Upper mask > none | -.82 | [-1.51, -.32] | -3.02** | .14 | [-.85, 1.20] | .26 |
| **Disgusted faces** | **---** | **---** | **---** | **---** | **---** | **---** |
| Lower mask > none | -.93 | [-1.61, -.40] | -3.24** | -.56 | [-1.87, .59] | -1.02 |
| Lower mask > upper | -.76 | [-1.60, -.19] | -2.55* | .009 | [-1.54, 1.57] | .016 |
| Upper mask > none | -.17 | [-.75, .42] | -.57 | -.55 | [-1.95, .59] | -1.00 |
| **Fearful faces** | **---** | **---** | **---** | **---** | **---** | **---** |
| Lower mask > none | -.34 | [-.98, .25] | -1.16 | -.47 | [-1.42, .28] | -1.16 |
| Lower mask > upper | -.47 | [-1.24, .17] | -1.44 | .17 | [-.65, 1.06] | .46 |
| Upper mask > none | .075 | [-.56, .69] | .25 | -.66 | [-1.57, .17] | -1.66 |
| **Sad faces** | **---** | **---** | **---** | **---** | **---** | **---** |
| Lower mask > none | -.89 | [-1.48, -.44] | -3.56*** | -1.06 | [-2.39, -.27] | -2.18* |
| Lower mask > upper | -.44 | [-.99, .027] | -1.82 | -1.33 | [-3.42, -.51] | -2.46* |
| Upper mask > none | -.48 | [-1.09, .00] | -1.87 | .31 | [-.72, 1.42] | .58 |
| **Surprised faces** | **---** | **---** | **---** | **---** | **---** | **---** |
| Lower mask > none | -.53 | [-1.03, -.078] | -2.23* | .26 | [-.58, 1.11] | .59 |
| Lower mask > upper | -.52 | [-1.07, -.064] | -2.23* | .010 | [-.83, .74] | .024 |
| Upper mask > none | -.013 | [-.54, .48] | -.053 | .27 | [-.53, 1.08] | .63 |
| *Note:* * *p* < .05. ** *p* < .01. *** *p* < .001. *CI* = .95. | | | | | | |

| Supplementary Table S7  *Misidentifying expressions: Face mask effects over sadness ratings.* | | | | | | |
| --- | --- | --- | --- | --- | --- | --- |
| --- | **Study 1** | | | **Study 2** | | |
| --- | *b* | *CI* | *z* | *b* | *CI* | *z* |
| **Angry faces** | **---** | **---** | **---** | **---** | **---** | **---** |
| Lower mask > none | .67 | [.24, 1.10] | 3.26** | .70 | [.097, 1.30] | 2.23* |
| Lower mask > upper | 1.28 | [.90, 1.66] | 6.54*** | 1.36 | [.75, 2.01] | 4.45*** |
| Upper mask > none | -.64 | [-1.03, -.25] | -3.18** | -.71 | [-1.30, -.13] | -2.30* |
| **Disgusted faces** | **---** | **---** | **---** | **---** | **---** | **---** |
| Lower mask > none | .20 | [-.13, .53] | 1.18 | -.84 | [-1.58, -.084] | -2.34* |
| Lower mask > upper | .28 | [-.051, .65] | 1.60 | -1.02 | [-1.69, -.33] | -2.86** |
| Upper mask > none | -.072 | [-.39, .27] | -.44 | .20 | [-.50, .92] | .54 |
| **Fearful faces** | **---** | **---** | **---** | **---** | **---** | **---** |
| Lower mask > none | .69 | [.27, 1.11] | 3.40*** | -.28 | [-.99, .46] | -.72 |
| Lower mask > upper | 1.17 | [.82, 1.56] | 5.96*** | .41 | [-.34, 1.15] | 1.07 |
| Upper mask > none | -.58 | [-.99, -.17] | -2.93** | -.71 | [-1.42, -.018] | -1.81 |
| **Happy faces** | **---** | **---** | **---** | **---** | **---** | **---** |
| Lower mask > none | -.58 | [-1.09, -.14] | -2.95** | -1.13 | [-2.21, -.61] | -3.21** |
| Lower mask > upper | -.54 | [-1.11, -.092] | -2.75** | -1.51 | [-3.03, -1.04] | -3.77*** |
| Upper mask > none | -.019 | [-.49, .42] | -.095 | .21 | [-.49, 1.00] | .56 |
| **Surprised faces** | **---** | **---** | **---** | **---** | **---** | **---** |
| Lower mask > none | -.15 | [-.48, .20] | -.85 | -.77 | [-1.53, -.13] | -2.20* |
| Lower mask > upper | -.13 | [-.43, .17] | -.84 | -.79 | [-1.22, -.41] | -3.92*** |
| Upper mask > none | -.006 | [-.36, .36] | -.037 | -.040 | [-.80, .64] | -.11 |
| *Note:* * *p* < .05. ** *p* < .01. *** *p* < .001. *CI* = .95. | | | | | | |

| Supplementary Table S8  *Misidentifying expressions: Face mask effects over surprise ratings.* | | | | | | |
| --- | --- | --- | --- | --- | --- | --- |
| --- | **Study 1** | | | **Study 2** | | |
| --- | *b* | *CI* | *z* | *b* | *CI* | *z* |
| **Angry faces** | **---** | **---** | **---** | **---** | **---** | **---** |
| Lower mask > none | -.15 | [-.60, .30] | -.82 | -1.05 | [-2.42, -.16] | -2.34* |
| Lower mask > upper | -.54 | [-1.07, -.14] | -2.92** | -1.84 | [-4.38, -1.14] | -3.60*** |
| Upper mask > none | .39 | [-.026, .88] | 2.14* | .85 | [-.057, 2.33] | 1.76 |
| **Disgusted faces** | **---** | **---** | **---** | **---** | **---** | **---** |
| Lower mask > none | -.16 | [-.48, .19] | -.95 | 1.21 | [.38, 2.00] | 2.94** |
| Lower mask > upper | -.35 | [-.73, .021] | -2.04* | .98 | [.16, 1.85] | 2.30* |
| Upper mask > none | .19 | [-.14, .56] | 1.11 | .15 | [-.65, .89] | .38 |
| **Fearful faces** | **---** | **---** | **---** | **---** | **---** | **---** |
| Lower mask > none | -1.36 | [-1.74, -1.02] | -7.82*** | -.28 | [-1.02, .53] | -.74 |
| Lower mask > upper | -3.07 | [-3.47, -2.67] | -14.94*** | -1.14 | [-1.88, -.35] | -2.91** |
| Upper mask > none | 1.78 | [1.42, 2.13] | 9.78*** | .88 | [.13, 1.64] | 2.33* |
| **Happy faces** | **---** | **---** | **---** | **---** | **---** | **---** |
| Lower mask > none | -.28 | [-.58, .012] | -1.90 | .003 | [-.65, .74] | .011 |
| Lower mask > upper | -.48 | [-.77, -.21] | -3.31*** | -.050 | [-.68, .56] | -.19 |
| Upper mask > none | .22 | [-.078, .52] | 1.48 | .073 | [-.56, .77] | .24 |
| **Sad faces** | **---** | **---** | **---** | **---** | **---** | **---** |
| Lower mask > none | -.62 | [-1.00, -.27] | -3.15** | -.56 | [-2.04, .59] | -1.11 |
| Lower mask > upper | -.63 | [-.95, -.30] | -3.93*** | -2.07 | [-8.29, -.85] | -2.94** |
| Upper mask > none | .057 | [-.34, .44] | .28 | 1.20 | [.20, 2.91] | 2.18* |
| *Note:* * *p* < .05. ** *p* < .01. *** *p* < .001. *CI* = .95. | | | | | | |
